# Lifestyle and incident dementia: A COSMIC individual participant data meta‐analysis

**DOI:** 10.1002/alz.13846

**Published:** 2024-04-27

**Authors:** Stephanie Van Asbroeck, Sebastian Köhler, Martin P. J. van Boxtel, Darren M. Lipnicki, John D. Crawford, Erico Castro‐Costa, Maria Fernanda Lima‐Costa, Sergio Luis Blay, Xiao Shifu, Tao Wang, Ling Yue, Richard B. Lipton, Mindy J. Katz, Carol A. Derby, Maëlenn Guerchet, Pierre‐Marie Preux, Pascal Mbelesso, Joanna Norton, Karen Ritchie, Ingmar Skoog, Jenna Najar, Therese Rydberg Sterner, Nikolaos Scarmeas, Mary Yannakoulia, Themis Dardiotis, Elena Rolandi, Annalisa Davin, Michele Rossi, Oye Gureje, Akin Ojagbemi, Toyin Bello, Ki Woong Kim, Ji Won Han, Dae Jong Oh, Stella Trompet, Jacobijn Gussekloo, Steffi G. Riedel‐Heller, Susanne Röhr, Alexander Pabst, Suzana Shahar, Nurul Fatin Malek Rivan, Devinder Kaur Ajit Singh, Erin Jacobsen, Mary Ganguli, Tiffany Hughes, Mary Haan, Allison E. Aiello, Ding Ding, Qianhua Zhao, Zhenxu Xiao, Kenji Narazaki, Tao Chen, Sanmei Chen, Tze Pin Ng, Xinyi Gwee, Qi Gao, Henry Brodaty, Julian Trollor, Nicole Kochan, Antonio Lobo, Javier Santabárbara, Patricia Gracia‐Garcia, Perminder S. Sachdev, Kay Deckers, for Cohort Studies of Memory in an International Consortium (COSMIC)

**Affiliations:** ^1^ Alzheimer Centrum Limburg Department of Psychiatry and Neuropsychology Mental Health and Neuroscience (MHeNs) Research Institute Maastricht University Maastricht The Netherlands; ^2^ Centre for Healthy Brain Ageing (CHeBA) Discipline of Psychiatry and Mental Health School of Clinical Medicine, University of New South Wales Sydney New South Wales Australia; ^3^ René Rachou Institute, Fiocruz Minas Belo Horizonte Minas Gerais Brazil; ^4^ Department of Psychiatry Federal University of São Paulo São Paulo Brazil; ^5^ Department of Geriatric Psychiatry Shanghai Mental Health Center, Shanghai Jiao Tong University School of Medicine Shanghai China; ^6^ Department of Psychiatry & Affective Disorders Center Ruijin Hospital, Shanghai Jiao Tong University School of Medicine Shanghai China; ^7^ Alzheimer's Disease and Related Disorders Center Shanghai Jiao Tong University Shanghai China; ^8^ Saul R. Korey Department of Neurology Albert Einstein College of Medicine Bronx New York USA; ^9^ Department of Epidemiology and Population Health Albert Einstein College of Medicine Bronx New York USA; ^10^ Department of Psychiatry and Behavioral Medicine Albert Einstein College of Medicine Bronx New York USA; ^11^ Inserm U1094, IRD UMR270, University Limoges, CHU Limoges, EpiMaCT ‐ Epidemiology of chronic diseases in tropical zone, Institute of Epidemiology and Tropical Neurology, OmegaHealth Limoges France; ^12^ Department of Neurology Amitié Hospital Bangui Central African Republic; ^13^ Institute for Neurosciences of Montpellier (INM), University of Montpellier, Inserm Montpellier France; ^14^ Institut du Cerveau Trocadéro Paris France; ^15^ Department of Psychiatry and Neurochemistry Neuropsychiatric Epidemiology Unit Institute of Neuroscience and Physiology, Sahlgrenska Academy, at the University of Gothenburg Gothenburg Sweden; ^16^ Centre for Ageing and Health (AGECAP), University of Gothenburg Gothenburg Sweden; ^17^ Region Västra Götaland, Sahlgrenska University Hospital, Psychiatry, Cognition and Old Age Psychiatry Clinic Gothenburg Sweden; ^18^ Department of Clinical Genetics Section Genomics of Neurodegenerative Diseases and Aging, Vrije Universiteit Amsterdam, Amsterdam UMC Amsterdam The Netherlands; ^19^ Department of Neurobiology Aging Research Center Care Sciences and Society, Karolinska Institute and Stockholm University Stockholm Sweden; ^20^ 1st Department of Neurology Aiginition Hospital, Medical School, National and Kapodistrian University of Athens Athens Greece; ^21^ Department of Neurology Taub Institute for Research on Alzheimer's Disease and the Aging Brain, Gertrude H. Sergievsky Center, Columbia University New York New York USA; ^22^ Department of Nutrition and Dietetics Harokopio University Athens Greece; ^23^ School of Medicine University of Thessaly Larissa Greece; ^24^ Golgi Cenci Foundation Milan Italy; ^25^ Department of Brain and Behavioral Sciences University of Pavia Pavia Italy; ^26^ Department of Psychiatry WHO Collaborating Centre for Research and Training in Mental Health, Neuroscience and Substance Abuse, University of Ibadan Ibadan Nigeria; ^27^ Department of Psychiatry College of Medicine University of Ibadan Ibadan Nigeria; ^28^ Department of Neuropsychiatry Seoul National University Bundang Hospital Seongnam Republic of Korea; ^29^ Department of Psychiatry Seoul National University College of Medicine Seoul Republic of Korea; ^30^ Department of Brain and Cognitive Sciences Seoul National University College of Natural Sciences Seoul Republic of Korea; ^31^ Workplace Mental Health Institute, Kangbuk Samsung Hospital, Sungkyunkwan University School of Medicine Seoul Republic of Korea; ^32^ Department of Internal Medicine section of Gerontology and Geriatrics Leiden University Medical Center Leiden the Netherlands; ^33^ Department of Public Health and Primary Care Leiden University Medical Center Leiden the Netherlands; ^34^ Institute of Social Medicine Occupational Health and Public Health Medical Faculty University of Leipzig Leipzig Germany; ^35^ Health and Ageing Research Team (HART), School of Psychology, Massey University Palmerston North Aotearoa New Zealand; ^36^ Global Brain Health Institute (GBHI), Trinity College Dublin Dublin Ireland; ^37^ Center for Healthy Ageing & Wellness (H‐CARE) Faculty of Health Sciences University Kebangsaan Malaysia Kuala Lumpur Malaysia; ^38^ Department of Psychiatry University of Pittsburgh School of Medicine Pittsburgh Pennsylvania USA; ^39^ Departments of Neurology, and Epidemiology University of Pittsburgh School of Medicine and School of Public Health Pittsburgh Pennsylvania USA; ^40^ Department of Graduate Studies in Health and Rehabilitation Sciences Bitonte College of Health and Human Services, Youngstown State University Youngstown Ohio USA; ^41^ Department of Epidemiology and Biostatistics School of Medicine, University of California San Francisco San Francisco California USA; ^42^ Columbia Aging Center and the Department of Epidemiology Mailman School of Public Health, Columbia University New York New York USA; ^43^ Institute of Neurology National Center for Neurological Disorders, National Clinical Research Center for Aging and Medicine, Huashan Hospital, Fudan University Shanghai China; ^44^ Center for Liberal Arts, Fukuoka Institute of Technology Higashi‐ku Fukuoka Japan; ^45^ Department of Physical Education Sports and Health Research Center Tongji University Shanghai China; ^46^ Graduate School of Biomedical and Health Sciences, Hiroshima University Hiroshima Japan; ^47^ Department of Psychological Medicine Gerontology Research Programme, Yong Loo Lin School of Medicine, National University of Singapore Singapore Singapore; ^48^ Department of Developmental Disability Neuropsychiatry Discipline of Psychiatry and Mental Health, University of New South Wales Sydney New South Wales Australia; ^49^ Department of Medicine and Psychiatry Universidad de Zaragoza Zaragoza Spain; ^50^ Instituto de Investigación Sanitaria Aragón (IIS Aragón) Zaragoza Spain; ^51^ CIBERSAM, Instituto de Salud Carlos III Madrid Spain; ^52^ Department of Public Health Universidad de Zaragoza Zaragoza Spain; ^53^ Neuropsychiatric Institute, The Prince of Wales Hospital Sydney New South Wales Australia

**Keywords:** age, dementia, dementia risk reduction, education, effect modification, ethnicity, individual participant data meta‐analysis, interaction, lifestyle, primary prevention, region, risk factor, risk personalization, sex, socioeconomic

## Abstract

**INTRODUCTION:**

The LIfestyle for BRAin Health (LIBRA) index yields a dementia risk score based on modifiable lifestyle factors and is validated in Western samples. We investigated whether the association between LIBRA scores and incident dementia is moderated by geographical location or sociodemographic characteristics.

**METHODS:**

We combined data from 21 prospective cohorts across six continents (*N* = 31,680) and conducted cohort‐specific Cox proportional hazard regression analyses in a two‐step individual participant data meta‐analysis.

**RESULTS:**

A one‐standard‐deviation increase in LIBRA score was associated with a 21% higher risk for dementia. The association was stronger for Asian cohorts compared to European cohorts, and for individuals aged ≤75 years (vs older), though only within the first 5 years of follow‐up. No interactions with sex, education, or socioeconomic position were observed.

**DISCUSSION:**

Modifiable risk and protective factors appear relevant for dementia risk reduction across diverse geographical and sociodemographic groups.

**Highlights:**

- A two‐step individual participant data meta‐analysis was conducted.
- This was done at a global scale using data from 21 ethno‐regionally diverse cohorts.
- The association between a modifiable dementia risk score and dementia was examined.
- The association was modified by geographical region and age at baseline.
- Yet, modifiable dementia risk and protective factors appear relevant in all investigated groups and regions.

## 1 BACKGROUND

Due to the aging of the population and lack of curative treatments, dementia prevalence is expected to increase from 55 million cases worldwide in 2019 to 139 million cases by 2050, further heightening the already large burden of disease associated with this group of conditions.^1^ However, evidence supporting the potential for dementia risk reduction by targeting modifiable risk and protective factors has been steadily accumulating.^2^, ^3^ About 40% of all dementia cases worldwide have been estimated to be potentially attributable to 12 modifiable risk and protective factors.^4^ Many of these factors also overlap with the World Health Organization's recommendations to reduce dementia risk.^5^

However, it is currently unclear whether dementia risk should be further personalized, or at least stratified, based on sociodemographic or other characteristics. Exploring risk stratification may lead to more accurate and inclusive risk estimates.^6^ For instance, the risk conferred by certain modifiable risk factors has been demonstrated to differ depending on the age of exposure. This has been the case for obesity, hypertension, and dyslipidemia which have been shown to be more strongly associated with dementia when their exposure occurs in mid‐life rather than in late life.^2^, ^7^, ^8^ However, little is known about whether other sociodemographic variables, such as sex, race and ethnicity, educational level, and socioeconomic position (SEP), or geographical location modify the association between modifiable risk and protective factors and incident dementia. Yet, this information may indicate where most preventive potential lies, which is important information for policy makers and individuals themselves.

To date, some studies from high income, Western countries have observed no effect modification by education or SEP,^9^, ^10^, ^11^ and studies looking at sex have shown varying results.^12^, ^13^, ^14^, ^15^ There are reports of continent‐level differences in the association between alcohol consumption and dementia risk^13^ and a stronger association between diabetes and cognitive decline in samples from Asian countries compared to samples from Europe, North America, and Australia with predominantly White participants.^16^ Yet, in general the research on potential (ethno‐) regional differences in associations between modifiable risk factors and future cognitive status is scarce. Therefore, risk personalization for all known modifiable risk and protective factors needs to be further investigated. In the case of dementia, composite modifiable risk scores such as the LIfestyle for BRAin Health (LIBRA) index^8^, ^17^ or the Australian National University Alzheimer's Disease Risk Index (ANU‐ADRI^18^) are also relevant to consider due to the syndrome's multifactor etiology.^19^, ^20^ These composite risk scores combine the presence or absence of multiple factors into one numeric value that expresses dementia risk. They are especially valuable as they can aid in the identification of individuals at high risk and may facilitate the implementation of dementia risk reduction guidelines into practice.^19^

The LIBRA index includes only modifiable risk (ie, physical inactivity, current smoking, obesity, hypertension, dyslipidemia, diabetes, depression, coronary heart disease [CHD], and chronic kidney disease [CKD]) and protective (ie, low‐to‐moderate alcohol consumption, healthy diet, high cognitive activity) factors.^8^, ^17^ The score has been well validated for predicting incident dementia in cohorts located in Western, high‐income countries.^9^, ^16^, ^17^, ^18^, ^19^, ^20^, ^21^ However, its predictive validity has not been explored in populations living in other regions. The current study aims to assess whether the association between the LIBRA index and incident dementia is moderated by sociodemographic characteristics, including age, sex, education, and SEP, or by geographical location.

## 2 METHODS

### 2.1 Contributing studies

In this individual‐participant data meta‐analysis, data from 21 cohorts, drawn from 17 countries across six continents, were used. All cohorts are part of the Cohort Studies of Memory in an International Consortium (COSMIC) collaboration.^21^ Details of the individual studies can be found in Table 1.

**Research in context**

1. **Systematic review**: The authors reviewed the current state‐of‐evidence regarding modifiable risk factors for dementia using traditional sources (eg, PubMed). Risk personalization or stratification beyond age has rarely been explored whereas doing so could identify groups with the most preventive potential and is necessary for inclusive and accurate risk estimation.
2. **Interpretation**: Our results suggest that modifiable factors, compiled in a modifiable dementia risk score, are associated with dementia risk regardless of sex, age, educational level, socioeconomic position, or broad geographical region. However, this association was even stronger in younger individuals (≤75 years) and in Asian cohorts compared to European cohorts.
3. **Future directions**: These findings need to be replicated. More data and research are needed particularly regarding geographical region. Cohort studies conducted in multiple regions would be ideal. Future work should also examine potential interactions between individual modifiable risk factors (rather than an index) and sociodemographic or environmental characteristics.

**TABLE 1:** Contributing studies, in alphabetical order.

Study	Abbreviation	Location (continent)	Year started	*n* included (%) of total sample
Bambui cohort study of ageing^22^	Bambui	Bambui, Brazil (South America)	1997	1336 (83.2)
China longitudinal aging study^23^	CLAS	China (Asia)	2011	1872 (57.7)
Einstein aging study^24^	EAS	New York City, NY, United States (North America)	1993	1022 (44.7)
Epidemiology of dementia in central Africa^25^	EPIDEMCA	Gamboma and Brazzaville, Republic of Congo (Africa)	2011	689 (70.0)
Enquête de Santé Psychologique—Risques, Incidence et Traitement^26^	ESPRIT	Montpellier, France (Europe)	1999	1983 (87.8)
The Gothenburg H70 Birth cohort study^27^	the H70 study	Gothenburg, Sweden (Europe)	1971	902 (73.9)
Hellenic longitudinal investigation of aging and diet^28^	HELIAD	Larissa and Marousi, Greece (Europe)	2010	1001 (48.1)
Invecchiamento Cerebrale in Abbiategrasso^29^	InveCe.Ab	Abbiategrasso, Italy (Europe)	2010	1107 (83.8)
Ibadan study of ageing^30^	ISA	Ibadan, Nigeria (Africa)	2003	1244 (57.9)
Korean longitudinal study on cognitive aging and dementia^31^	KLOSCAD	South Korea (Asia)	2009	5109 (75.0)
Leiden 85‐plus study^32^	Leiden 85+	Leiden, the Netherlands (Europe)	1997	485 (81.0)
Leipzig longitudinal study of the aged^33^	LEILA 75+	Leipzig, Germany (Europe)	1997	891 (70.4)
The longitudinal study on neuroprotective model for healthy longevity^34^	LRGS TUA	Malaysia (Asia)	2012	1006 (43.2)
Maastricht aging study^35^	MAAS	South Limburg, the Netherlands (Europe)	1993	1643 (61.8)
Monongahela‐Youghiogheny healthy aging team^36^	MYHAT	Allegheny County, PA, United States (North America)	2006	1653 (86.1)
Sacramento area Latino study on aging^37^	SALSA	Sacramento area, CA, United States (North America)	1998	1471 (82.2)
Shanghai aging study^38^	SAS	Shanghai, China (Asia)	2010	1657 (43.2)
Sasaguri Genkimon Study^39^	SGS	Sasaguri, Japan (Asia)	2011	1050 (39.9)
Singapore longitudinal ageing study II^40^	SLAS II	Singapore (Asia)	2003	1433 (43.8)
Sydney memory and ageing study^41^	MAS	Sydney, Australia (Oceania)	2005	900 (86.8)
Zaragoza dementia depression project^42^	ZARADEMP	Zaragoza, Spain (Europe)	1994	3226 (67.2)

**FIGURE 1:**
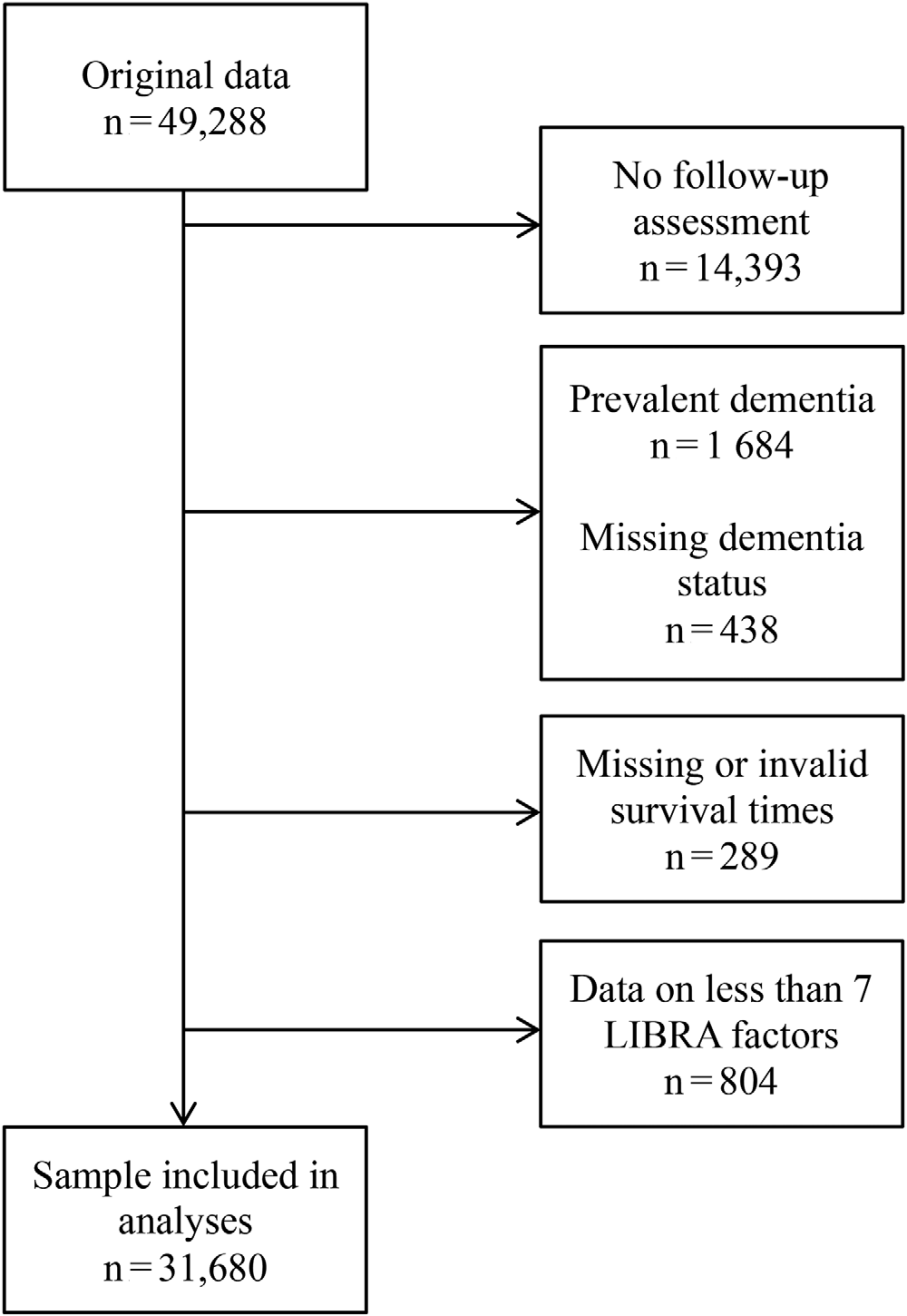
Flow chart of sample selection. LIBRA, LIfestyle for BRAin Health (index).

Exclusion criteria were the following: prevalent dementia, missing information on dementia status or length of follow‐up, no available follow‐up assessment, or insufficient data on modifiable risk and protective factors (ie, fewer than seven out of the 12 LIBRA factors, Figure 1).

This study was approved by the University of New South Wales Human Research Ethics committee (HC17292 and HC220222). All individual cohorts had previously received local ethical approval (see Material S1).

### 2.2 Sociodemographic characteristics and geographical location

Age and sex data were available in all cohorts. Educational level was operationalized as total years of formal education. If unavailable, categories of educational attainment were converted to total years of formal education after consultation with local study coordinators. For a stratified analysis, years of formal education were categorized into low (<6 years of formal education), intermediate (6 to 11 years of formal education) and high (≥12 years of formal education). SEP was categorized as low, middle, or high within each cohort, based on several alternative measures, predominantly income and occupation, to maximize harmonization potential. See Material S2 for the sociodemographic characteristics’ harmonization protocols. Geographical location was defined as the continent where the cohort was sampled. Within nearly every cohort the sample was predominantly homogeneous in terms of broad racial and ethnic group. Race and ethnicity, and geographical location, overlapped almost entirely: non‐Hispanic White in Europe and Australia, Asian in Asia, and non‐Hispanic Black in Africa. The cohorts in North America included one predominantly non‐Hispanic White (MYHAT), one fully Hispanic (SALSA, no further specification on White or Black but mostly of Mexican origin), and one mixed (EAS: non‐Hispanic White, Hispanic White, non‐Hispanic Black, and Hispanic Black). The only cohort in South America (Bambui) was from Brazil, a country known to have a very complex and hard to define racial and ethnic makeup.^43^

### 2.3 Modifiable risk and protective factors: LIBRA score

Modifiable risk factors were summarized in the LIBRA score, a weighted compound score that combines the presence or absence of 12 modifiable risk and protective factors for dementia.^8^, ^17^ Each risk and protective factor has an assigned weight based on the factor's relative risk for dementia from published meta‐analyses.^8^, ^17^ Based on the presence or absence of the factor, these weights (see Material S2) are summed to yield the total LIBRA score, ranging from −5.9 to +12.7. Higher scores indicate a less favorable combination ofrisk factors and a higher risk for dementia. The LIBRA index has been extensively validated to predict brain damage (ie, white matter hyperintensities volume on neuroimaging), cognitive decline, incident cognitive impairment, and dementia risk in various population‐based studies.^10^, ^17^, ^44^, ^45^, ^46^, ^47^, ^48^

Here, the presence or absence of each factor was determined for every individual, based on the harmonization protocols described in Material S2. If possible, missing information at baseline was augmented with information at follow‐up assessments. At least seven of the 12 factors had to be available for an individual to be included in the analyses (Figure 1). When not all factors were known, the LIBRA score was calculated with the available factors and rescaled to the full theoretical range (ie, when all 12 factors would be present) using the standard min‐max normalization formula given as follows in Equation (1),

(1) $$\mathrm{LIBR}A_{\mathrm{scal}\mathrm{ed}}=\;\frac{\mathrm{LIBR}A_{\mathrm{crude}}-a}{b-a}\times \left(12.7-\left(-5.9\right)\right)+\left(-5.9\right)$$

where *a* and *b* are the lower and upper limits, respectively, of the LIBRA score with the available factors.

### 2.4 Dementia incidence

Determination of incident dementia varied across studies. Twelve studies used Diagnostic and Statistical Manual of Mental Disorders (DSM) III or IV criteria.^49^, ^50^ A few cohorts employed other assessment methods, such as the Clinical Dementia Rating (CDR^51^), the Mini‐Mental State Examination (MMSE^52^), or a self‐reported diagnosis. It was not possible to distinguish between different etiologies, as brain imaging, biomarkers, or autopsy data were not available. The harmonization protocol can be found in Material S2.

### 2.5 Statistical analyses

A two‐step individual participant data meta‐analysis approach was used. Potential associations between sociodemographic characteristics and LIBRA score at baseline were examined by calculating the cohort‐specific differences in mean LIBRA score between the sociodemographic groups. Next, these mean differences were pooled using random‐effects meta‐analysis resulting in pooled mean differences and their 95% confidence intervals (CIs).

The LIBRA score was also compared between geographical locations. Here, mean LIBRA scores were pooled per continent and compared using a Cochran's Q test. The *I* ^2^ statistic was used to assess heterogeneity between studies. Meta‐regression was used to confirm the results from the Cochran's Q test and estimate the mean difference in LIBRA score between continents.

Cox proportional hazard regression analysis was used to assess the association between the LIBRA score and dementia incidence in each cohort separately, resulting in hazard ratios (HRs) and their 95% CIs. This was done for the continuous LIBRA score as well as for cohort‐specific LIBRA score tertiles. In all analyses, age was used as the time scale and dementia was treated as the failure event. Survival time was defined as the age at study entry until the age at the date of dementia diagnosis (if reported, otherwise calculated as the mid‐point between waves) or study exit (ie, date of last interview or date of death, whichever came first). Three different models were run, controlling for the following variables: Model 1 = crude model (age only); Model 2 = Model 1 + sex and years of formal education (main model); and Model 3 = Model 2 + SEP. Model 3 was defined as a separate model because data on SEP were not available in a significant portion of the sample. Also because of this, Model 2 is regarded as the main model. As dementia's pathology develops over many years before diagnosis, additional analyses were carried out in which survival time within each cohort was split up into an early follow‐up (≤5 years after baseline) and a late follow‐up (>5 years after baseline). This allows us to distinguish between incident cases that were likely already in the preclinical stage at baseline, and cases that developed later. Each Cox regression analysis had to have at least five events (ie, incident dementia cases) per variable in the model (ie, LIBRA score + control variables).^53^ The proportional hazard assumption was assessed by testing of Schoenfeld residuals.^54^ One cohort (Gothenburg H70 Birth cohort study [the H70 study]) showed non‐proportional hazards. As there is no straightforward remedy for this, without compromising the ability to compare and pool the results of analyses over all cohorts, this cohort was still included in the analyses. However, an additional sensitivity analysis without the H70 study was carried out. To examine the moderating effect of the sociodemographic variables described earlier, Cox regression analyses, stratified by these variables, were executed. Baseline age was stratified into individuals ≤75 versus older, as LIBRA was initially designed for capturing modifiable dementia risk in people aged 40 to 75 years and has been shown to perform less well after the age of 75.^7^, ^44^ All cohort‐specific HRs were pooled using random effects meta‐analysis. Cochran's Q statistic for subgroup differences was used to test the potential moderating effect of the selected sociodemographic variables and geographical location on the association between LIBRA and dementia incidence with an alpha level of 0.10.^55^ The *I* ^2^ statistic was used to assess heterogeneity between studies. Additionally, meta‐regression was used to examine potential sources of heterogeneity (including proportion of females, median age, median follow‐up time, number of available LIBRA factors in a cohort, continent, and gross‐domestic product per capita of the country where the cohort was based^56^). All tests were carried out two‐sided with an alpha level of 0.05 unless indicated otherwise. All analyses were conducted with Stata 17.0 (StataCorp, College Station, Texas, United States).

## 3 RESULTS

In total, 31,680 eligible individuals from 21 cohorts across six continents were included (mean age range at baseline: 52 to 85 [SD range across cohorts: 0 to 16], 58% female, Figure 1). A total of 2330 incident dementia cases were recorded during an overall median of 5.0 years of follow‐up (range: 1.0 to 2.4 years). Of these, 1671 dementia cases were diagnosed ≤5 years after baseline, and 659 cases were diagnosed later (>5 years after baseline). Detailed, cohort‐specific population characteristics can be found in Table 2. The cohort‐specific distribution of individual LIBRA factors can be consulted in Material S2. Included individuals were compared with those who were excluded. Across the cohorts, included individuals were typically younger and had more years of formal education. They also tended to be less likely to have conditions such as hypertension, diabetes, or depression, but were more likely to have dyslipidemia. A full comparison can be found in Material S3.

**TABLE 2:** Cohort‐specific population characteristics.

Cohort	Age, median (IQR)	Female sex, *n* (%)	Years of formal education, median (IQR)	LIBRA index, median (IQR)	Median follow‐up, years	Dementia incidence, *n* (%)
Bambui	67 (10)	829 (62)	3 (4)	2.9 (3.5)	11.0	173 (12)
CLAS	71 (13)	1008 (54)	9 (8)	−0.1 (3.9)	1.0	162 (9)
EAS	77 (8)	635 (62)	14 (4)	1.3 (4.2)	3.8	154 (11)
EPIDEMCA	72 (10)	407 (59)	0 (3)	1.6 (4.4)	1.9	36 (5)
ESPRIT	72 (7)	1168 (59)	11 (3)	2.0 (3.4)	11.5	210 (11)
the H70 study	70 (8)	672 (75)	8 (4)	3.2 (3.5)	11.7	146 (16)
HELIAD	72 (7)	597 (60)	6 (7)	3.0 (3.3)	3.0	56 (6)
InveCe.Ab	72 (2)	598 (54)	5 (3)	1.0 (3.7)	8.1	111 (10)
ISA	72 (11)	639 (51)	0 (5)	−2.0 (4.3)	5.5	136 (11)
KLOSCAD	69 (10)	2898 (57)	9 (6)	−2.2 (3.4)	5.5	252 (5)
Leiden 85+	85 (0)	315 (65)	6 (3)	1.9 (3.7)	5.0	64 (13)
LEILA 75+	80 (7)	653 (73)	12 (1)	3.8 (4.5)	4.1	214 (22)
LRGS TUA	67 (8)	525 (52)	6 (7)	1.5 (3.8)	5.0	48 (5)
MAAS	51 (26)	796 (48)	11 (4)	−0.7 (3.5)	12.4	62 (4)
MYHAT	78 (12)	1028 (62)	12 (2)	0.9 (4.4)	6.1	105 (6)
SALSA	69 (10)	856 (58)	7 (9)	2.0 (4.5)	7.7	103 (7)
SAS	71 (12)	900 (54)	12 (6)	−0.3 (4.0)	5.2	167 (10)
SGS	72 (9)	583 (56)	12 (3)	−2.9 (2.9)	2.0	5 (0.5)
SLAS II	65 (10)	929 (65)	6 (7)	0.6 (3.0)	4.2	11 (1)
MAS	78 (7)	488 (54)	11 (5)	2.4 (3.8)	5.8	106 (12)
ZARADEMP	70 (13)	1784 (55)	8 (3)	−3.1 (2.6)	4.5	138 (4)
**Total**	**71 (11)**	**18,308 (58)**	**9 (7)**	**0.2 (4.8)**	**5.0**	**2330 (7)**

*Note*: LIBRA theoretical range: −5.9 to +12.7, missing data: CLAS: sex: *n* = 2, years of formal education: *n* = 6. Expansions of cohort name abbreviations are presented in Table 1.

Abbreviations: IQR, interquartile range; LIBRA, LIfestyle for BRAin Health.

### 3.1 LIBRA index across sociodemographic strata and geographical location

**FIGURE 2:**
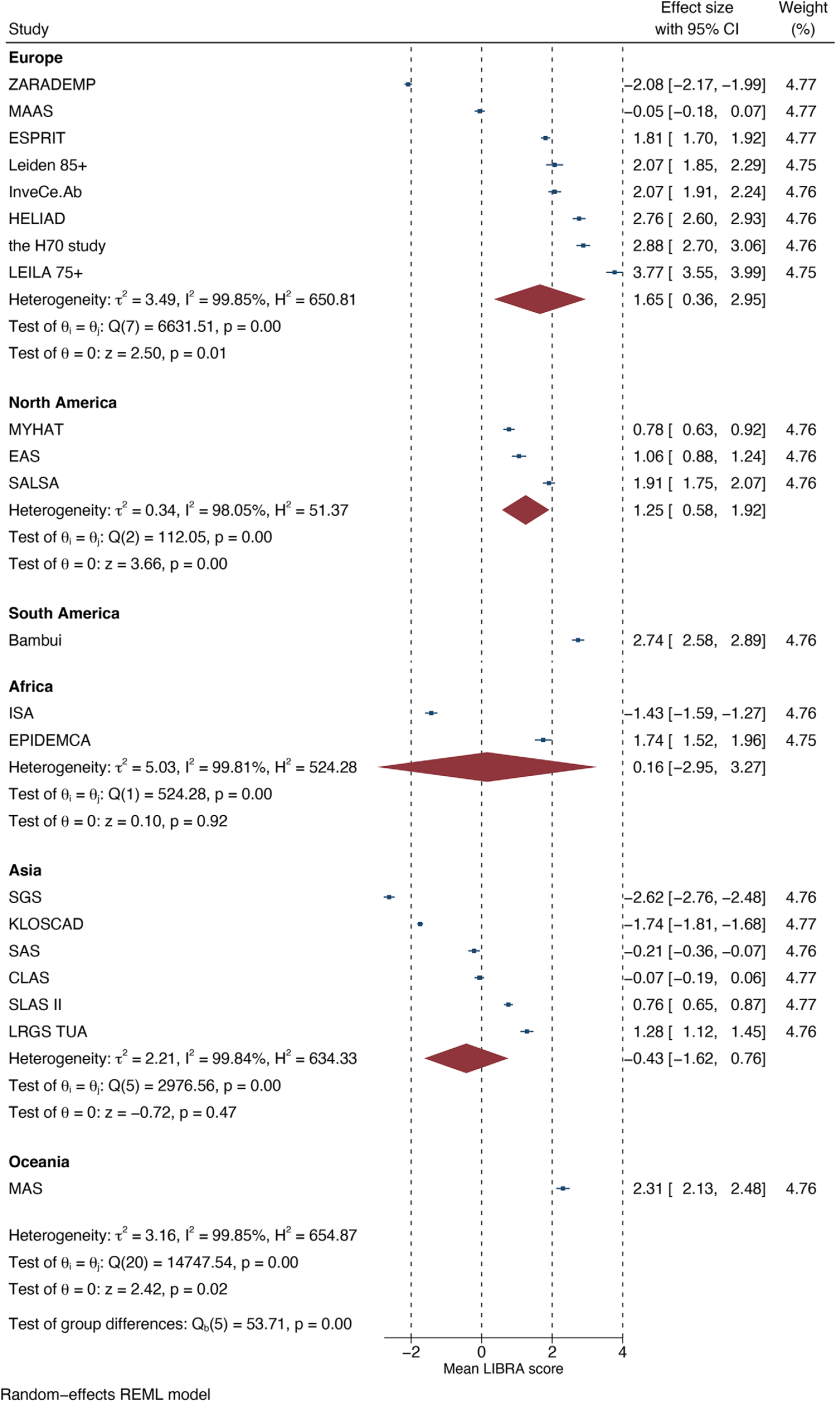
Mean LIBRA score by cohort and continent. Expansions of cohort name abbreviations are presented in Table 1. CI, confidence interval; LIBRA, LIfestyle for BRAin Health.

Potential baseline differences in mean LIBRA scores between sociodemographic strata and geographical location were assessed. Mean (95% CI) LIBRA scores were 0.5 (0.27 to 0.72) points higher (worse) in older individuals (≤75 vs >75 years old). LIBRA scores were also higher in individuals with fewer years of formal education or a lower SEP. For education, compared to 6 to 11 years, those with <6 years were 0.52 points higher and those with ≥12 years were 0.58 points lower (all *p* < 0.05). For SEP, compared to those with an intermediate SEP, those with a lower SEP were 0.39 points higher and those with a high SEP were 0.39 points lower (all *p* < 0.05). Mean LIBRA scores did not differ significantly between males and females. Forest plots of individual study data can be found in Material S4. LIBRA scores were also compared based on the geographical location of the cohort (Figure 2). This showed large heterogeneity both within and between continental regions. LIBRA scores were on average 2.1 (95% CI  = −3.8 to −0.3) points lower in Asian cohorts compared to European cohorts (*p* = 0.020).

### 3.2 LIBRA and dementia incidence

**FIGURE 3:**
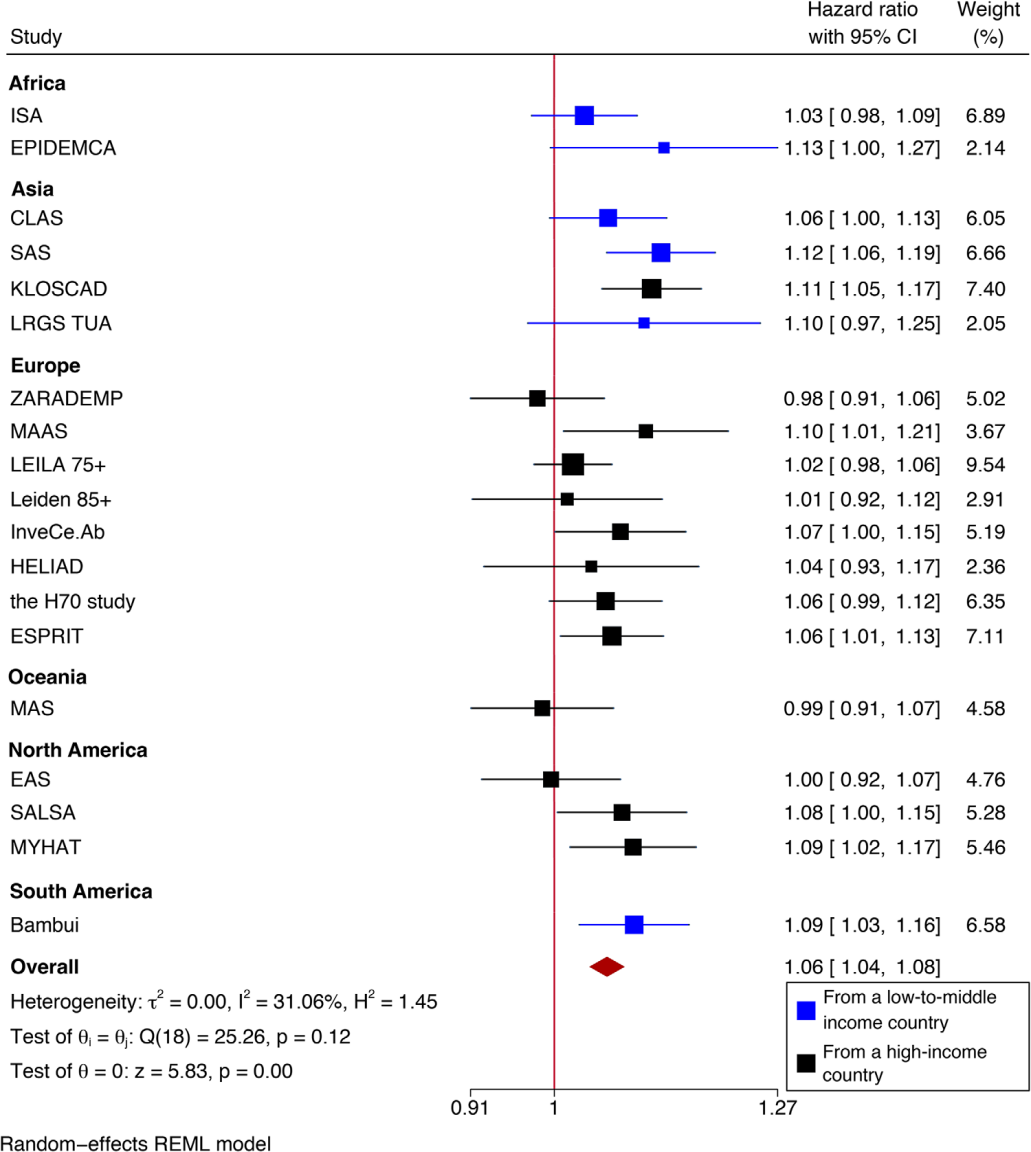
Hazard ratio for incident dementia per one‐point increase in LIBRA score. Model 2 (main model), controlled for age (time scale), sex, and years of formal education. Expansions of cohort name abbreviations are presented in Table 1. CI, confidence interval; HR, hazard ratio; LIBRA, LIfestyle for BRAin Health.

On a continuous scale, a one‐point increase in the LIBRA score was significantly associated with a higher risk for dementia (HR = 1.06, 95% CI = 1.04 to 1.08) in main Model 2 (Figure 3 and Table 3). Per one SD increase in LIBRA (over all participants in all cohorts combined: SD = 3.26), this translates to HR = 1.21 (95% CI = 1.14 to 1.29). Heterogeneity was limited (*I* ^2^ = 31.1%). The hazard for dementia was 33% higher in the highest LIBRA tertile compared to the lowest (Model 2, Table 3).

**TABLE 3:** Association between LIBRA score and dementia incidence.

LIBRA score	Model 1, HR (95% CI)	Model 2, HR (95% CI)	Model 3, HR (95% CI)
**Across complete follow‐up**			
Continuous^a^	**1.07 (1.05 to 1.09)**	**1.06 (1.04 to 1.08)**	**1.05 (1.03 to 1.08)**
Lowest tertile	Reference	Reference	Reference
Middle tertile	**1.19 (1.03 to 1.40)**	1.13 (0.99 to 1.30)	1.08 (0.90 to 1.30)
Highest tertile	**1.44 (1.26 to 1.65)**	**1.33 (1.18 to 1.50)**	**1.28 (1.10 to 1.49)**
**Early follow‐up (≤ 5 years after baseline)**			
Continuous^a^	**1.07 (1.04 to 1.10)**	**1.06 (1.03 to 1.09)**	**1.06 (1.02 to 1.09)**
Lowest tertile	Reference	Reference	Reference
Middle tertile	1.16 (0.98 to 1.38)	1.08 (0.93 to 1.26)	1.04 (0.87 to 1.24)
Highest tertile	**1.41 (1.19 to 1.68)**	**1.30 (1.13 to 1.51)**	**1.30 (1.08 to 1.56)**
**Late follow‐up (> 5 years after baseline)**			
Continuous^a^	**1.07 (1.04 to 1.10)**	**1.06 (1.03 to 1.09)**	**1.07 (1.03 to 1.10)**
Lowest tertile	Reference	Reference	Reference
Middle tertile	**1.23 (1.03 to 1.47)**	1.20 (1.00 to 1.43)	1.15 (0.92 to 1.44)
Highest tertile	**1.47 (1.22 to 1.77)**	**1.40 (1.16 to 1.69)**	**1.43 (1.14 to 1.81)**

*Note*: Model 1 controlled for age (time scale). Model 2 (main model): Model 1 + sex and years of formal education. Model 3: Model 2 + socioeconomic position.

Abbreviations: CI, confidence interval; HR, hazard ratio; LIBRA, LIfestyle for BRAin Health.

^a^ per one‐point increase.

When examining early and late follow‐up, the LIBRA index was associated with dementia incidence, both in individuals who developed dementia within 5 years (HR = 1.06, 95% CI = 1.03 to 1.09, Model 2) and in individuals who developed dementia after more than 5 years (also HR = 1.06, 95% CI = 1.03 to 1.09, Model 2).

### 3.3 Potential moderating effect of sociodemographic characteristics and geographical location on the association between LIBRA and incident dementia

**FIGURE 4:**
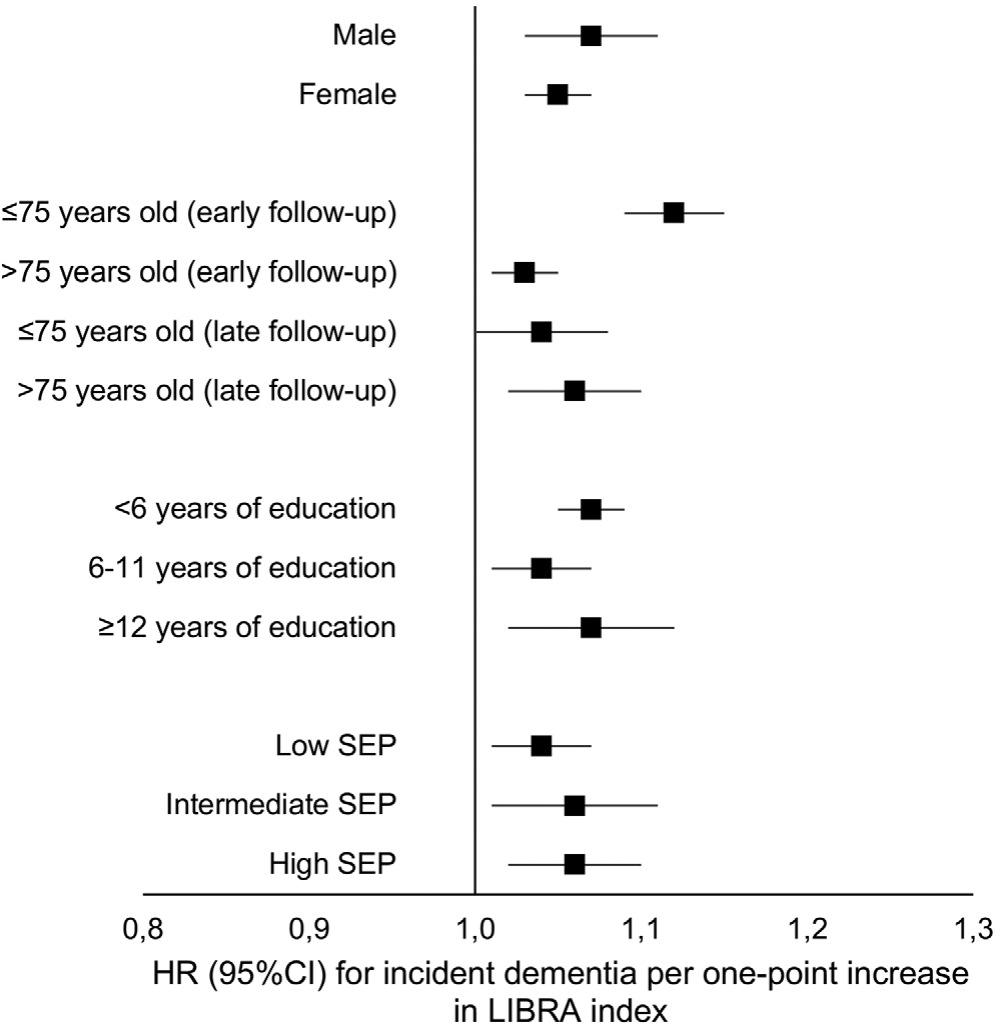
Hazard ratio for incident dementia per one‐point increase in LIBRA score per sociodemographic group. Model 2 (main model), controlled for age (time scale), sex, and years of formal education. CI, confidence interval; HR, hazard ratio; LIBRA, LIfestyle for BRAin Health; SEP, socioeconomic position.

Potential interactions between LIBRA, sociodemographic characteristics and geographical location were explored using meta‐analysis and Cochran's Q statistic for subgroup differences (Figure 4 and Material S5), as well as meta‐regression (Material S6).

The association between LIBRA and dementia incidence did not differ between males and females, regardless of timing during the follow‐up. Further, years of formal education and SEP did not interact with LIBRA. When comparing the association between LIBRA and incident dementia among younger (≤75 years old) and older (>75 years old) individuals across the entire follow‐up time, evidence for an interaction was observed. With higher LIBRA scores, the risk increase was larger in younger individuals (1.08, 95% CI = 1.05 to 1.11) than in older individuals (1.04, 95% CI = 1.02 to 1.07, *p*_interaction _= 0.035). Meta‐regression confirmed that median age at baseline was indeed a significant moderator. However, this interaction was only apparent in the early follow‐up period (Cochran's Q test, Model 2 younger: 1.12, 95% CI = 1.09 to 1.15, vs older: 1.03, 95% CI = 1.01 to 1.06, *p*_interaction _< 0.001). When considering late follow‐up cases (occurring after at least 5 years), baseline age did not alter the association between LIBRA and dementia incidence (Cochran's Q test, Model 2 younger: 1.04, 95% CI = 1.00 to 1.08, vs older: 1.06, 95% CI = 1.02 to 1.10, *p*_interaction _= 0.545). Meta‐regression analysis confirmed these results: median baseline age was a significant moderator in the early follow‐up period but not in the late follow‐up period. Lastly, across the entire follow‐up period, the association between LIBRA and incident dementia was found to be stronger in Asian cohorts (1.10, 95% CI = 1.07 to 1.14) compared to European cohorts (1.04, 95% CI = 1.02 to 1.07, *p*_interaction _= 0.069). However, when examining this difference for early and late follow‐up separately, there was no significant effect modification by geographical region (early follow‐up Asian cohorts: 1.09, 95% CI = 1.06 to 1.13), vs early follow‐up European cohorts: 1.05, 95% CI = 1.00 to 1.10, *p*_interaction _= 0.599; late follow‐up Asian cohorts: 1.14, 95% CI = 0.98 to 1.31, vs late follow‐up European cohorts: 1.06, 95% CI = 1.01 to 1.10, *p*_interaction _= 0.610). These findings were confirmed in the meta‐regression analysis. Full results on the stratified meta‐analysis can be found in Material S5 and detailed results of the meta‐regression can be found in Material S6. A sensitivity analysis omitting the H70 study cohort because of non‐proportional hazards was also run. The results were similar and can be seen in Material S7.

## 4 DISCUSSION

In this individual participant data meta‐analysis, modifiable dementia risk profiles from diverse ethno‐regional groups were examined in 21 prospective cohort studies from 17 countries. An unfavorable modifiable risk profile was associated with an increased risk for dementia, and this association was stronger with younger baseline ages (≤75 years), specifically for dementia cases occurring in the first 5 years of follow‐up. Across the entire follow‐up time, the association between LIBRA index and incident dementia appeared stronger in Asian compared to European cohorts. However, this interaction disappeared when considering early and late follow‐up cases separately. Importantly, no interactions between modifiable risk profiles and other sociodemographic variables (ie, sex, years of formal education, SEP) were observed.

First, this study examined the association between modifiable risk factors and dementia incidence at a global scale. So far, the link between modifiable risk factors and dementia risk has been predominantly based on studies from high income Western countries, while estimates from low‐ and middle‐income countries (LMICs) were lacking. Yet, the largest increase in dementia prevalence is projected for LMICs.^1^, ^57^ The current study included cohorts from six LMICs (Bambui from Brazil, CLAS, and SAS from China, LRGS TUA from Malaysia, EPIDEMCA from the Republic of Congo, and ISA from Nigeria; see Table 1 for expansions of cohort name abbreviations). Our findings suggest that modifiable risk factors are relevant for dementia risk reduction in different parts of the world and might have important implications for global efforts to reduce dementia risk.^1^, ^58^

Across the entire follow‐up period, the association between the LIBRA score and incident dementia was stronger in Asian cohorts compared to European cohorts. Previous research suggests that Asian individuals show greater susceptibility to type 2 diabetes, cardiovascular disease, stroke, and associated mortality than White individuals, even when exposure to modifiable risk factors for these outcomes (eg, elevated body mass index or serum triglycerides) is similar.^55^, ^56^, ^57^ However, this stronger association between LIBRA and dementia risk within the Asian cohorts was not observed when considering early and late follow‐up cases separately. This may be because of insufficient power to detect in an interaction, especially within late follow‐up. In early follow‐up, however, sample size of Asian cohorts was still large. Upon further investigation, a suppressor effect between follow‐up time and geographical region was noticed, in which controlling for follow‐up resulted in a larger estimated effect of geographical region, as well as a significant effect of follow‐up time (with longer follow‐up time being associated with a larger HR). Without controlling for region, no significant effect of follow‐up time was noticed as Asian cohorts tended to have a shorter follow‐up time compared to European and American cohorts. We therefore contemplate that the unobserved interaction between LIBRA and geographical region in early follow‐up is mostly due to the fact that region and follow‐up time suppress each other when they are not both included in the model. When splitting survival time, the effect of follow‐up time is modelled less well. Other work looking at ethnic and/or regional differences in susceptibility is limited. One 10/66 Dementia Research Group population‐based study compared multiple dementia risk prediction models (including modifiable risk factors) for their predictive validity in LMICs (China and several Latin American countries).^59^ All assessed prediction models were developed based on Western, and predominantly White, populations. They found that ANU‐ADRI and the Brief Dementia Screening Indicator (BDSI^60^) replicated well in the studied LMICs, but the Cardiovascular Risk Factors, Aging and Dementia (CAIDE^61^) risk score did not. It remains unclear whether there are ethno‐regional differences in susceptibility to modifiable risk factors for dementia. More research is needed.

No interactions were observed between the LIBRA score and sex, years of formal education, or SEP. A healthy lifestyle or favorable risk profile thus appears to be associated with a lower risk for dementia to a similar degree in all assessed groups. It is important to note that individuals with more years of formal education or a higher SEP tended to have a more favorable risk factor profile. The lack of effect modification by education or SEP is in line with a limited number of earlier findings, originating from Western countries.^9^, ^10^, ^11^ A previous COSMIC study examining the potential interaction between sex and specific modifiable risk factors also concluded that there was generally no interaction,^12^ but other studies have shown varying results.^13^, ^14^, ^15^

Age interacted with the LIBRA score, which is in line with earlier findings, demonstrating that LIBRA is (more) predictive of incident dementia in individuals in mid‐to‐early‐late life compared to individuals in late‐late life. For example, the Cambridge City over‐75s cohort study found no association between the LIBRA score—nor its individual risk factors—and dementia risk in individuals over 85 years old,^7^ whereas an association between LIBRA and incident dementia is consistently reported in younger populations.^10^, ^17^, ^46^ Similar results were observed in another study using data from the European population‐based DESCRIPA study.^44^ From a life course perspective, one may indeed expect more benefit from adhering to a healthy lifestyle at a younger age, before the pathological process of decades of exposure to risk factors has caused extensive damage.^62^ Interestingly, this interaction was specifically observed for early dementia cases that occurred within the first 5 years after baseline. In other words, when a dementia diagnosis occurs within 5 years after the risk profile assessment (when the likelihood for reverse causality is much larger), the risk profile is more strongly associated with dementia risk in the younger (≤75 years) individuals. Potentially, the older the age at baseline, the more time might be needed for effects of the risk factor profile on dementia risk to become noticeable, as exposure to risk factors before that time has played a larger role. However, this result may also be related to a larger proportion of the sample being Asian in early follow‐up compared to late follow‐up. Asian cohorts tended to be younger and were associated with a larger HR compared to European cohorts. Overall, this demonstrates again that it is important to consider the timing of these diagnoses.

The current results hint at universal prevention initiatives. However, this study only considered a small part of the many steps there are between the establishment of a modifiable risk factor (or clustering of multiple risk and protective factors) and the actual evidence‐based implementation of effective strategies aimed at altering the exposure to them (for example, this is also dependent on risk factor prevalence or cost‐effectiveness).

### 4.1 Strengths and limitations

Strengths of this study include its overall sample size and sufficiently high number of incident dementia cases for finding associations. Due to the relatively large amount of individual participant data, we were able to use more refined and elaborate analysis methods. Nonetheless, it is important to consider that during the process of data harmonization, information can get lost which may result in bias. The global context of this study is uncommon and leads to good external validity. However, data from South America, Africa, Oceania, and certain Asian regions (especially the Middle East and Central Asia) were still limited. Therefore, our results on potential effect modification by geographical region need to be replicated. Furthermore, potential differences based on geographical location, as well as race and ethnicity, should ideally be examined. Due to the nature of the data here, with a very high level of overlap between these two variables, doing so was not reasonable. Instead, we had to focus on one variable and geographical location was chosen because it was readily available for all cohorts and meant a larger sample size. Race and ethnicity were not adequately assessed in several of the cohorts that we aimed to include. Further, we were not able to control for apolipoprotein E (*APOE*) *ε*4 allele carriership and information on treatments (eg, for managing dyslipidemia or hypertension) was not always available. Another limitation lies in the fact that we looked at the presence of risk factors at just one timepoint during the lives of these individuals. Also, the studied outcome was incident dementia diagnosis, which is not equivalent to the onset of underlying disease, and may also depend on the healthcare system, testing procedures, and so on. Additionally, for some cohorts many individuals were excluded as they did not meet the preset inclusion criteria. They often differed from the included individuals (see Material S3). In general, younger, more educated, and healthier individuals were included. Included individuals were more likely to have dyslipidemia compared to excluded individuals. Loss to follow‐up or dropout due to worse health (ie, multiple comorbidities) is a recurrent observation in longitudinal studies but cannot be remediated easily. Indeed, most people were excluded because they only had cross‐sectional data. This could have resulted in an underestimation of observed associations.

Taken together, these results suggest that a “brain‐healthy” lifestyle is associated with a lower risk for dementia years later, regardless of geographical location or sociodemographic characteristics. However, exposure to these modifiable risk and protective factors varied greatly across the ethno‐regionally diverse cohorts. Further research towards risk stratification and personalization is needed and should focus on a life course approach, examining the association between a brain‐healthy lifestyle and dementia risk at different timepoints during the entire life.^63^

## CONFLICT OF INTEREST STATEMENT

AL has received financial support to attend scientific meetings from Janssen. PG has received financial support to attend scientific meetings from Lundbeck, Esteve, Nutrición Médica, Angelini, and Neuraxpharm. DD reports grants from Shanghai Municipal Science and Technology Major Project (2018SHZDZX01) and ZJ LAB, National Natural Science Foundation of China (82173599, 81773513), Scientific Research Plan Project of Shanghai Science and Technology Committee (17411950701, 17411950106), and National Project of Chronic Disease (2016YFC1306402); all payments were made to the institution. QZ reports grants from the National Chronic Disease Project (2016YFC1306402), Shanghai Science and Technology Municipality (17411950106, 2018SHZDZX03, 17411950701), National Natural Science Foundation of China (82071200, 81773513), Shanghai Hospital Development Center (SHDC2020CR4007), and MOE Frontiers Center for Brain Science (JIH2642001/028); all payments were made to the institution. NS declares personal fees from NIH, grants from Novo Nordisk (not related to current manuscript). All other authors do not have any conflicts of interest to declare. Author disclosures are available in the supporting information.

## CONSENT STATEMENT

All participants provided informed consent.

## Supporting information

Supporting Information

Supporting Information

Supporting Information

Supporting Information

Supporting Information

Supporting Information

Supporting Information

Supporting Information

## Data Availability

Data were provided by the contributing studies on the understanding and proviso that the relevant study leaders be contacted for further use of their data and additional formal data sharing agreements be made. Researchers can apply to use COSMIC data by completing a COSMIC Research Proposal Form available from https://cheba.unsw.edu.au/consortia/cosmic/research‐proposals.
