# Supplementary material for: Lifestyle and incident dementia: A COSMIC individual participant data meta‐analysis
Source: Alzheimers Dement. 2024 Apr 27;20(6):3972–86. [Article in Italian] doi: 10.1002/alz.13846 (PMC11180928; doi:10.1002/alz.13846)
Supplement: Supplementary file 1 — Supporting Information [file ALZ-20-3972-s001.docx]

**Supplementary material 1: Ethical approvals**

| **Study** | **Institutional Review Board** |
| --- | --- |
| Bambui | Ethics Boards of the Fundação Oswaldo Cruz (Fiocruz) in Rio de Janeiro and the Instituto René Rachou of the Fundação Oswaldo Cruz (Fiocruz) in Belo Horizonte, Brazil (14/2007 - CEPSH-CpqRR) |
| CLAS | Ethics Committee of Shanghai Mental Health Center (2011-YJ-14) |
| EAS | Albert Einstein College of Medicine Institutional Review Board (Approval#1996-175) |
| EPIDEMCA | Ethical committees, supervised by Ministry of Public Health in CAR (8/UB/FACSS/CSCVPER/11) and the Comité d’Ethique de la Recherche en Sciences de Santé in ROC (00000204/DGRST/CERSSA), approved the study protocol, as well as the “Comité de la Protection des Personnes Sud-Ouest Outre-Mer” in France (SOOM4/CE/3). |
| ESPRIT | Ethics committee (CCPPRB) of the Kremlin Bicetre hospital (n° registered 99-28) |
| the H70 study | The study was approved by the Regional Ethical Review Board, and all methods were performed in accordance with the Helsinki Declaration. |
| HELIAD | Institutional Ethics Review Board of the University of Thessaly (ΒΕΥ846Ψ8Ν2-32Π) |
| InveCe.Ab | Ethics Committee of the University of Pavia (#3/2009) |
| ISA | UI/UCH Research Ethics Committee – UI/IRC/02/07P |
| KLOSCAD | Institutional Review Board of Seoul National University Bundang Hospital, Korea (IRB No. B-0912/089-010) |
| Leiden 85+ | Leiden University Medical Center, 1996 |
| LEILA 75+ | Ethics committee of the University of Leipzig (C7 79934700) |
| MAAS | Ethics committee of Maastricht University Medical Centre (MEC05-107) |
| LRGS TUA | National University of Malaysia (UKM) research ethics committee (reference: UKM PPI/111/8/JEP-2019-024), and was also approved by the Malaysian National Medical Research and Ethics Committee at the Malaysian Ministry of Health [reference: KKM/NIHSEC/P19-1689(12)] |
| MYHAT | University of Pittsburgh Human Research Protection Office (formerly Institutional Review Board, IRB). Approval # PRO16030244 |
| SALSA | University of California, San Francisco Human Research Protection Program Institutional Review Board (IRB#10-00243) |
| SAS | Medical Ethics Committee of Huashan Hospital, Fudan University, Shanghai, China (approval number: HIRB2009-195) |
| SGS | Institutional Review Board of the Institute of Health Science, Kyushu University (IHS-2010-22) |
| SLAS II | National University of Singapore Institutional Review Board (Reference Code: 04-140) |
| MAS | University of New South Wales Human Research Ethics Committee (approval #14327) |
| ZARADEMP | Ethics committee of the Zaragoza University Hospital (CEICA # CP16/2012) |
