# Supplementary material for: Lifestyle and incident dementia: A COSMIC individual participant data meta‐analysis
Source: Alzheimers Dement. 2024 Apr 27;20(6):3972–86. [Article in Italian] doi: 10.1002/alz.13846 (PMC11180928; doi:10.1002/alz.13846)
Supplement: Supplementary file 2 — Supporting Information [file ALZ-20-3972-s002.docx]

**Supplementary material 2: Harmonization protocol, operationalization of the variables and distribution of the modifiable risk and protective factors in all cohorts**

1. **LIfestyle for BRAin health (LIBRA) score weights^1^**

| **LIfestyle for BRAin health (LIBRA) factors** | **Weight** |
| --- | --- |
| Physical inactivity | +1.1 |
| Current smoker | +1.5 |
| Obesity | +1.6 |
| Hypertension | +1.6 |
| Dyslipidemia | +1.4 |
| Diabetes | +1.3 |
| Depression | +2.1 |
| Chronic kidney disease | +1.1 |
| Coronary heart disease | +1.0 |
| Low-to-moderate alcohol consumption | -1.0 |
| High cognitive activity | -3.2 |
| Healthy diet | -1.7 |

1. **Years of formal education**

For all cohorts besides the ones listed here, years of formal education were available. When only categories of educational attainment were available, these were converted to years of formal education as listed below.

| **Study** | **Category** | **Years of formal education** |
| --- | --- | --- |
| ESPRIT | None | 0 |
|  | Primary | 5 |
|  | 1st to 4th year senior school/higher primary | 9 |
|  | Short technical or professional | 11 |
|  | 5th year to upper 6th form | 12 |
|  | Long technical or professional | 12 |
|  | Higher education including higher technical education | 15 |
| MAAS | Elementary education | 6 |
|  | Lower vocational education | 9 |
|  | Intermediate secondary education | 9 |
|  | Intermediate vocational education | 10 |
|  | Higher secondary education | 11 |
|  | Higher vocational education | 15 |
|  | University education | 16 |
|  | Scientific education | 17 |
| EPIDEMCA | Never attended school | 0 |
|  | Schooled but never completed | 3 |
|  | Primary completed | 6 |
|  | Secondary completed | 13 |
|  | Tertiary or higher education | 16 |
| Leiden 85+ | None | 0 |
|  | Elementary education | 6 |
|  | Lower vocational education | 9 |
|  | Intermediate secondary education | 9 |
|  | Intermediate vocational education | 10 |
|  | Higher secondary education | 11 |
|  | Higher vocational education | 15 |
|  | Scientific education | 17 |
| ISA | No education (0 years) | 0 |
|  | Primary education (1-6 years) | 5 |
|  | Secondary education (7-12 years) | 10 |
|  | Tertiary education (13+ years) | 14 |

1. **Socioeconomic position (SEP)**

| **Study** | **Socioeconomic position measure(s) used** |
| --- | --- |
| Bambui | Three categories of income relative to minimum salary (<2x minimum salary: low, <4x minimum salary: middle, ≥ 4x minimum salary: high) |
| CLAS | Primary occupation |
| EAS | Current income (below poverty level: low, poverty level up to 2x poverty level: middle, >2x poverty level: high) |
| EPIDEMCA | / |
| ESPRIT | / |
| the H70 study | Primary occupation |
| HELIAD | 1. Primary occupation, 2. If occupation unavailable: tertiles of the surface area of the housing divided by the number of people in household |
| InveCe.Ab | Primary occupation |
| ISA | 1. Socioeconomic status (validated measure based on an inventory of household and personal items^2^, four categories were provided: low: low, low-average: middle, high-average: high, high: high). 2. If socioeconomic status unavailable: ISCO occupation |
| KLOSCAD | 1. Total household income (in US dollars; three predefined categories); 2. If income unavailable: disadvantaged or not (based on receiving medical aid): when disadvantaged classify as low SEP |
| Leiden 85+ | / |
| LEILA 75+ | Primary occupation |
| MAAS | 1. Income: divided by tertiles, 2. If income unavailable: occupation and occupation partner |
| LRGS TUA | 1. If current income is not enough for basic needs: low, 2. Household income: divided by tertiles |
| MYHAT | / |
| SALSA | Household gross income: divided by tertiles |
| SAS | Three predefined levels of income |
| SGS | / |
| SLAS II | 1. Primary occupation, 2. If occupation unavailable: three types of housing (1-3 rooms: low, 4-5 rooms: middle, higher end house: high) |
| MAS | Primary occupation |
| ZARADEMP | Occupation |

Occupations were classified according to the European socio-economic classification^3^ with class 1, 2 and 3 categorized as high; class 4, 5 and 6 as middle; and class 7, 8 and 9 as low SEP. If an individual did not have an occupation and no other data was available, SEP would be missing. Abbreviations: International Standard Classification of Occupations (ISCO), socioeconomic position (SEP).

1. **Dementia incidence**

| **Study** | **Criteria** |
| --- | --- |
| Bambui | Mini-Mental State Examination (MMSE) score cut-off point 13/14 appropriate for Brazilian populations with low schooling^4,5^ |
| CLAS | Clinical diagnosis or Clinical Dementia Rating (CDR) ≥ 1 if no clinical assessment^6^ |
| EAS | Diagnostic and Statistical Manual of Mental Disorders (DSM) IV^7^ |
| EPIDEMCA | DSM-IV^7^ |
| ESPRIT | Standardized interview by a neurologist incorporating cognitive testing, with diagnoses validated by an independent panel of expert neurologists |
| the H70 study | DSM-III-R^8^ |
| HELIAD | DSM-IV^7^ |
| InveCe.Ab | DSM-IV^7^ |
| ISA | Identification based on the 10-Word Delayed Recall Test^9^ and the Clinician Home-based Interview to assess Function^10^ |
| KLOSCAD | DSM-IV^7^ |
| Leiden 85+ | Self-reported history |
| LEILA 75+ | DSM-IV^7^ |
| MAAS | DSM-IV^7^ |
| LRGS TUA | CDR ≥1^6^ |
| MYHAT | Combination of CDR ≥1^6^ and consensus diagnosis by investigators (n=100) |
| SALSA | First screened using the Modified Mini-Mental State Examination (3MSE^11^), and the Spanish and English Verbal Learning Test (SEVLT^12^). Those scoring poorly and a random sample of 20% of all participants did a neuropsychological test battery including the Spanish English Neuropsychological Assessment Scales (SENAS^13^) and The Informant Questionnaire on Cognitive Decline in the Elderly (IQCODE^14^). These were used for a clinical consensus diagnosis. |
| SAS | DSM-IV consensus diagnosis^7^ |
| SGS | Self-reported history of dementia |
| SLAS II | DSM-IV^7^ |
| MAS | DSM-IV consensus diagnosis^7^ |
| ZARADEMP | DSM-IV^7^ |

1. **Smoking status**

Smoking status was either current smoker or non-smoker based on self-report.

1. **Obesity**

Obesity was defined according to the World Health Organization definition (body mass index (BMI) ≥ 30 kg/m^2^) and was calculated using the participants’ (self-reported) weight and height.^15^

1. **Alcohol consumption**

Individuals were categorized in two groups based on drinking up to seven standard units or 70 g of pure alcohol per week, or more than this.^16^

| **Study** |  |
| --- | --- |
| Bambui | / |
| CLAS | / |
| EAS | Drinks per week calculated from past year categorical consumption of beer, wine and liquor (mid-point of each category was used) |
| EPIDEMCA | Average number of units per week |
| ESPRIT | Grams of alcohol per week |
| the H70 study | Categories of grams of alcohol consumed per week:  Less than or equal to 100 g/week: ≤ 7 standard units/week  More than 100 g/week: >7 units/week |
| HELIAD | Grams of alcohol per week |
| InveCe.ab | / |
| ISA | / |
| KLOSCAD | Standard drinks per week |
| Leiden 85+ | Standard drinks per week |
| LEILA 75+ | Average drinks per week |
| MAAS | Standard units per week |
| LRGS TUA | / |
| MYHAT | Calculated total number of drinks per week |
| SALSA | Number of drinks per week |
| SAS | / |
| SGS | / |
| SLAS II | Categorical frequency of alcoholic drinks:  Never or rarely: ≤ 7 standard units/week  More than 1 per month but less than 1 per week: ≤ 7 standard units/week  More than 1 per week but less than 1 per day: ≤ 7 standard units/week  1-2 per day: ≤ 7 standard units/week  3 or more per day: > 7 standard units/week |
| MAS | Calculated total number of drinks per week |
| ZARADEMP | Calculated total number of drinks per week |

1. **Chronic kidney disease**

An estimated glomerular filtration rate (eGFR) < 60 ml/min/1.73m^2^ was considered as chronic kidney disease. If creatinine levels were provided, the recommended Chronic Kidney Disease Epidemiology Collaboration (CKD- EPI) equation was used to calculate eGFR.^17^

| **Study** |  |
| --- | --- |
| Bambui | / |
| CLAS | / |
| EAS | / |
| EPIDEMCA | / |
| ESPRIT | / |
| the H70 study | / |
| HELIAD | 1. eGFR (calculated using creatinine), 2. If eGFR unavailable: renal insufficiency diagnosis |
| InveCe.Ab | Chronic kidney disease (confirmed by a geriatrician during clinical assessment) |
| ISA | / |
| KLOSCAD | 1. eGFR (calculated using creatinine), 2. If eGFR unavailable: medical history |
| Leiden 85+ | eGFR calculated using creatinine |
| LEILA 75+ | / |
| MAAS | Currently with condition |
| LRGS TUA | Self-reported kidney failure |
| MYHAT | / |
| SALSA | / |
| SAS | eGFR |
| SGS | Self-reported history of chronic kidney disease or prostate disease |
| SLAS II | eGFR (calculated using the Modification of Diet in Renal Disease study (MDRD) formula^18^) |
| MAS | 1. Long standing kidney disease diagnosed by doctor, 2. eGFR (calculated using creatinine) |
| ZARADEMP | / |

1. **Cognitive activity**

All cognitive activities an individual engages in were counted (one point per activity) and this score was divided into tertiles per cohort. Individuals in the highest tertile were considered cognitively active. Individuals in the middle and lowest tertile were considered cognitively inactive. Included activities per cohort are listed here.

| **Study** |  |
| --- | --- |
| Bambui | 1 point for each activity they engage in at least “less than once a month”: Visits from children who do not live with you, visits from relatives, social gatherings with people from work, visits from friends, religious services, association meetings. Final score: 0-6. |
| CLAS | Used the Social support rating scale (SSRS ^19^): 1 point if individual at least occasionally attends organized group activities, 2 points if individual takes initiative to attend and is active with organized group activities, 1 point if individual does not “stay away from family, lives alone”, 1 point if individual has at least one intimate friend, 1 point if person does “other activities”. Final score: 0-5. |
| EAS | 1 point for each activity they do at least 30 min/week: reading newspaper, reading books, crosswords, tv, using computer, using e-mail; 1 point for each activity they at least 30 min/month: knitting, writing, music, singing, museums, theater, board games, cards games, learning, support groups and other activities (open input). Final score: 0-17 |
| EPIDEMCA | 1 point for each: has friends, plays games, participates in child’s education, participation in ceremonies, associations or societies (occasionally or regularly). Final score: 0-4. |
| ESPRIT | 1 point for each activity they do at least “sometimes”: artistic activity, sports; further add 1 point for each activity they do at least 1-2h per week: home repairs, gardening, knitting or sewing, walking (categories are: less than 1h, 1-2 h, more than 2h). Final score: 0-6 |
| the H70 study | 1 point for each:  club membership, member of retirement club, member of another club;  visits from children, visits from grandchildren, participating in club meetings, bingo, hobbies, taking part in course activities, going to church, watching or listening to church tv or church radio (at least once in three months);  visits from neighbors (at least sometimes);  listening to the radio, watching tv, reading the newspaper;  reading books (at least monthly). Final score: 0-16 |
| HELIAD | 1 point for each activity they do at least 30 min: driving, watching tv, household work, reading or using computer, dancing, sitting or standing with friends, holiday (if yes: 1 point). Final score 0-7. |
| InveCe.Ab | 1 point if they engage at least “sometimes” in this activity: play cards, read magazines, reading books, going to cinema or theatre, attend courses, charity, involved in associations, artistic activities, knit, other leisure activities. Final score: 0-10. |
| ISA | Limited data: cognitively active if both family and community participation |
| KLOSCAD | / |
| Leiden 85+ | / |
| LEILA 75+ | 1 point for each activity they engage in “at least monthly”: seeing children or relatives, seeing or chatting to someone in community, seeing someone from neighborhood or friends; further add 1 point if they attend religious meetings at least occasionally and 1 point if they attend at least occasionally social meetings. Final score: 0-5. |
| MAAS | 1 point for each activity done at least 30 min/week: tv, reading, associations, sports, games, light exercise, housework, pastimes, learning new stuff, meeting up with friends or relatives. Final score: 0-10. |
| LRGS TUA | 1 point for each activity that is done at least “often”: drive, cycle or ride bike, work in farm, eat out in restaurant, simple mechanical repairing (at least seldom), visit relatives or friends, sew or do embroidery, fishing or business or catering, recreational activities, household chores, attend or organize parties, brain games, reading, religious classes or seminars, shopping, member of association, watch tv, managing household finances, educational activities, community-based activities, use computer or laptop or iPad, stock market or further studies, prepare food at home, vacation (at least seldom), babysitting, having a pet (if yes: 1 point). Final score: 0-24. |
| MYHAT | 1 point for each activity from the Florida Cognitive Activities Scale (FCAS^20^) they engage in at least once per month: Final score: 0-25. |
| SALSA | 1 point per activity they engage in it at least 30 min: yardwork, house repairs, baking, walking or walking around neighborhood, swim or work out, housework (light or heavy), cooking meals, dancing, hunting, camping or boating, golf or moderate exercise, driving car. Final score 0-10. |
| SAS | Provided cognitive activity score^21^ |
| SGS | Cognitively active if individual provided a positive response to both the following questions. “Do you regularly read newspapers?” and “Do you regularly read books/magazines?” |
| SLAS II | Each “yes” answer is 1 point: walked in past 2 weeks, cycled in past 2 weeks, worked in garden in past 2 weeks, do light or heavy household work, play cards, play computer games, anything cognitively stimulating, attend church, visit cinema or restaurant or sports event, day trip, join citizen club activities, participate in social activities, karaoke singing, hobbies. Final score: 0-14. |
| MAS | Each “yes” answer is 1 point: charity, television, radio, newspaper, magazines, books, classical music, games, museums etc., internet, artistic pastime, second language, new sport, new pastime, evening class, new certificate course, new uni degree. Final score: 0-17. |
| ZARADEMP | Cognitively active if individual did not decrease or cease neither social events or hobbies |

1. **Coronary heart disease**

| **Study** |  |
| --- | --- |
| Bambui | Myocardial infarction or angina |
| CLAS | Myocardial infarction or angina |
| EAS | Self-reported myocardial infarction, angina, coronary artery bypass or angioplasty |
| EPIDEMCA | / |
| ESPRIT | Cardiovascular disease |
| the H70 study | Myocardial infarction or angina |
| HELIAD | Coronary disease or myocardial infarction |
| InveCe.Ab | Coronary heart disease (confirmed by a geriatrician during clinical assessment) |
|  |  |
| ISA | Angina |
| KLOSCAD | History of/or current angina or myocardial infarction |
|  |  |
| Leiden 85+ | Myocardial infarction or angina |
| LEILA 75+ | Self-reported coronary heart disease |
| MAAS | Self-reported heart disease |
| LRGS TUA | Self-reported heart disease |
| MYHAT | Myocardial infarction, bypass surgery |
| SALSA | Myocardial infarction, angina |
| SAS | Heart disease (including coronary heart disease, valvular heart disease, cardiomyopathy, heart failure, heart rhythm problems) self-reported and confirmed by medical records |
| SGS | Self-reported history of cardiovascular disease |
| SLAS II | Self-reported heart disease |
|  |  |
| MAS | Myocardial infarction, angina |
| ZARADEMP | Diagnosis of myocardial infarction or angina using EURODEM Risk Factor Questionnaire and medical records ^22^ |

1. **Depression**

Current depression, defined as listed below. Meeting one of the criteria was sufficient.

| **Study** |  |
| --- | --- |
| Bambui | General Health Questionnaire-12^23^: score ≥ 5 |
| CLAS | Geriatric Depression Scale (GDS30^24^) score >10, depressive disorder (DSM based on structured clinical interview), self-report |
| EAS | Major depression (DSM-IV), anti-depressant use, GDS15^25^ score ≥ 6 |
| EPIDEMCA | Geriatric Mental State version B3 (GMS-AGECAT^26^) |
| ESPRIT | Current major depressive episode (MINI neuropsychiatric  Examination^27^), Center for Epidemiologic Studies Depression scale  (CES-D^28^) score ≥ 16 |
| the H70 study | Diagnosis based on DSM criteria, if unavailable: Montgomery Asberg Depression rating scale^29^ score ≥ 7 |
| HELIAD | GDS15^25^ score ≥ 6, anti-depressant use |
| InveCe.Ab | Anti-depressant use, GDS15^25^ score ≥ 6, criteria-based diagnosis by physician or psychologist (including medication, GDS score and CES-D items)^30^ |
| ISA | GDS15^25^ score ≥ 6 |
| KLOSCAD | Korean version of the Geriatric Depression Scale (GDS-K^31^) score ≥ 16 |
| Leiden 85+ | GDS15^25^ score ≥ 6, anti-depressant use |
| LEILA 75+ | DSM-IV criteria based on structured clinical interview^7^, CES-D^28^ score ≥ 16 |
| MAAS | Dutch version of the depression subscale of the Symptom Checklist 90. Participants in highest quartile have clinically relevant depression^32^ |
| MYHAT | Anti-depressant use |
| LRGS TUA | / |
| SALSA | CES-D^28^ score ≥ 16, anti-depressant use |
| SAS | CES-D^28^ score ≥ 16 |
| SGS | Self-reported history of depression |
| SLAS II | GDS15^25^ score ≥ 6 |
| MAS | GDS15^25^ score ≥ 6, anti-depressant use |
| ZARADEMP | Geriatric mental state B (GMS-B) followed by AGE-CAT system^26^ |

1. **Diabetes**

Fasting blood glucose ≥ 7 mmol/L or >125 mg/dL was considered diabetic. Meeting one of the criteria was sufficient.

| **Study** |  |
| --- | --- |
| Bambui | Fasting blood glucose, on treatment with insulin or antidiabetics |
| CLAS | Fasting blood glucose, self-reported diagnosis |
| EAS | History of diagnosis |
| EPIDEMCA | Fasting blood glucose, non-fasting blood glucose (>200 mg/dL), self-reported diagnosis |
| ESPRIT | Fasting blood glucose, on treatment with insulin or antidiabetics, self-reported diagnosis |
| the H70 study | Glucose levels, self-reported diagnosis, on treatment with insulin or antidiabetics |
| HELIAD | On treatment with insulin or antidiabetics, non-fasting glucose (>200 mg/dL), if no measurements are available: history of diagnosis |
| InveCe.Ab | Fasting blood glucose, on treatment with insulin or antidiabetics, history of diagnosis |
| ISA | Self-reported diagnosis |
| KLOSCAD | Self-reported diagnosis (current or history) |
| Leiden 85+ | Fasting blood glucose, on treatment with insulin or antidiabetics, HbA1c ≥ 6.5%, if no measurements available: medical history (by GP or pharmacist) |
| LEILA 75+ | Self-reported diagnosis |
| MAAS | Self-reported diagnosis or starting medication age 40+ |
| LRGS TUA | Fasting blood glucose, self-reported diagnosis |
| MYHAT | Self-reported diagnosis, on treatment with insulin or antidiabetics |
| SALSA | Self-reported diagnosis, fasting blood glucose, on treatment with insulin or antidiabetics |
| SAS | Self-reported history of diabetes |
| SGS | Self-reported history of diabetes |
| SLAS II | Fasting blood glucose, self-reported diagnosis |
| MAS | Fasting blood glucose, diagnosis confirmed by doctor |
| ZARADEMP | Diagnosis using EURODEM Risk Factor Questionnaire and medical records^22^ |

1. **Healthy diet**

A healthy diet was defined as adhering to the World Health Organization’s recommendation of consuming at least 400 g (i.e. 5 portions) of fruits and vegetables per day (excluding potatoes, sweet potatoes, cassava, etc.).^33^

| **Study** |  |
| --- | --- |
| Bambui | / |
| CLAS | Type of diet was available: meat-based 🡪 not adhering to a healthy diet, vegetable-based and meat- and vegetable-based 🡪 adhering to a healthy diet (proxy) |
| EAS | / |
| EPIDEMCA | Frequency of consumption of various fruits and vegetables was available. This was summed and divided in sex-specific tertiles. Those in the highest tertile were considered to adhere to a healthy diet. |
| ESPRIT | / |
| the H70 study | / |
| HELIAD | Summed portions of fruit and vegetable intake per day |
| InveCe.Ab | Vegetable consumption at least 3 times per week and fruit consumption at least three times per week: adherence to healthy diet  If frequency of fruit or vegetable is lower: no adherence healthy diet (proxy) |
| ISA | Summed servings of fruit and vegetables per day |
| KLOSCAD | / |
| Leiden 85+ | / |
| LEILA 75+ | / |
| MAAS | / |
| LRGS TUA | Summed weight of fruit and vegetable intake per day |
| MYHAT | / |
| SALSA | / |
| SAS | / |
| SGS | / |
| SLAS II | Summed portions of fruit and vegetable intake per day |
| MAS | Summed weight of fruit and vegetable intake per day |
| ZARADEMP | / |

1. **Dyslipidemia**

Individuals were categorized as having dyslipidemia based on a self-reported diagnosis and/or total cholesterol levels (dyslipidemia when total cholesterol ≥ 200 mg/dL) and/or medication use. If total cholesterol was not available, low density lipoprotein cholesterol (LDL-C) levels were considered (dyslipidemia if LDL-C ≥ 130 mg/dL (3.4 mmol/L)). If LDL-C was unavailable, triglyceride levels were considered (dyslipidemia if triglycerides >150 mg/dl (1.7 mmol/L)). If triglyceride levels were unavailable, high density lipoprotein cholesterol (HDL-C) levels were used (<35 mg/dL). Cut-offs are based on second report of the National Cholesterol Education Program (NECP).^34^

| **Study** | **Available data** |
| --- | --- |
| Bambui | Triglycerides |
| CLAS | Total cholesterol, LDL-C, HDL-C, triglycerides, medical history |
| EAS | Total cholesterol, LDL-C, HDL-C, triglycerides |
| EPIDEMCA | Total cholesterol |
| ESPRIT | Total cholesterol, hypolipemic medication use, self-reported elevated cholesterol |
| the H70 study | Total cholesterol, LDL-C, HDL-C, if measures unavailable: previously categorized variable based on triglycerides/HDL-C ≥ 5 or LDL-C ≥ 3.5 mmol/L or on lipid-lowering drugs |
| HELIAD | Total cholesterol, LDL-C, triglycerides, HDL-C, self-reported elevated cholesterol, hypolipemic medication use |
| InveCe.Ab | Total cholesterol, LDL-C, triglycerides, HDL-C, hypolipemic medication use |
| ISA | / |
| KLOSCAD | Currently with dyslipidemia, history of dyslipidemia |
| Leiden 85+ | Total cholesterol, LDL-C, triglycerides, HDL-C, hypolipemic medication use |
| LEILA 75+ | / |
| MAAS | Self-reported elevated cholesterol, self-reported hypolipemic medication use |
| LRGS TUA | Total cholesterol, HDL-C, LDL-C, triglycerides, self-reported hypolipemic medication use, self-reported elevated cholesterol |
| MYHAT | Total cholesterol, self-reported elevated cholesterol, hypolipemic medication use |
| SALSA | Total cholesterol, hypolipemic medication use, LDL-C, triglycerides |
| SAS | A predefined hyperlipemia variable was provided, which was based on measurements of total cholesterol, LDL-C, HDL-C and triglycerides |
| SGS | Self-reported history of dyslipidemia |
| SLAS II | Total cholesterol, self-reported elevated cholesterol, LDL-C, HDL-C, triglycerides |
| MAS | History, total cholesterol, TG, HDL-C, LDL-C |
| ZARADEMP | Self-reported elevated cholesterol |

1. **Hypertension**

Someone was considered to have hypertension when seated systolic blood pressure ≥ 140 mmHg or diastolic blood pressure ≥ 90 mmHg, if anti-hypertensive medication was used or if the participant self-reported having hypertension.

| **Study** |  |
| --- | --- |
| Bambui | Blood pressure measurements, history of hypertension |
| CLAS | Medical record of hypertension, blood pressure measurements |
| EAS | Blood pressure measurements (mean of two measurements), self-reported ever diagnosis |
| EPIDEMCA | Blood pressure measurements, self-reported ever diagnosis |
| ESPRIT | Blood pressure measurements (mean of two measurements), anti-hypertensive use, self-reported history |
| the H70 study | Blood pressure measurements, self-reported hypertension |
| HELIAD | Anti-hypertensive medication use, history of hypertension |
| InveCe.Ab | Anti-hypertensive medication use, supine blood pressure 170-180 mmHg and history of hypertension, supine blood pressure >180 mmHg |
| ISA | Self-reported hypertension, blood pressure measurements |
| KLOSCAD | Anti-hypertensive medication use, blood pressure measurements (mean of three measurements |
| Leiden 85+ | Anti-hypertensive medication use, blood pressure measurements, history of hypertension |
| LEILA 75+ | Blood pressure measurements |
| MAAS | Blood pressure measurements (mean of five measurements), anti-hypertensive medication use |
| LRGS TUA | Self-reported hypertension, blood pressure measurements |
| MYHAT | Blood pressure measurements, self-reported hypertension, anti-hypertensive medication use |
| SALSA | Self-reported hypertension, blood pressure measurements (mean of two measurements), anti-hypertensive medication use |
| SAS | Self-reported physician diagnosed history of hypertension |
| SGS | Blood pressure measurements, self-reported history of hypertension |
| SLAS II | Blood pressure measurements, self-reported hypertension |
| MAS | Blood pressure measurements (mean of two measurements), diagnosis confirmed by a doctor, anti-hypertensive medication use |
| ZARADEMP | Diagnosis using EURODEM Risk Factor Questionnaire and medical records^22^ |

1. **Physical activity**

Generally, we considered an individual to be physically active when meeting the World Health Organization recommendation of at least 150 minutes of moderate intensity physical activity or 75 minutes of vigorous intensity physical activity per week.^35^

| **Study** |  |
| --- | --- |
| Bambui | Considered physically active if participating in exercise lasting 20-30 minutes during leisure time at least three times a week |
| CLAS | Considered physically active if meeting any of the following criteria: sports at least 4 times per week, does physical labor |
| EAS | (Time in moderate intensity physical activity during last 2 weeks + time in vigorous physical activity in 2 weeks x 2) / 2 |
| EPIDEMCA | Time spent doing physical activity per week |
| ESPRIT | Considered physically active if meeting any of the following criteria: sports regularly or often, walking at least 1h/day, gardening at least 1h/day, home repair at least 1h/day |
| the H70 study | Considered physically active if meeting any of the following criteria: engages in moderate or intense physical activity in Summer and Winter, does daily walking, at least 150 minutes walking per week |
| HELIAD | Calculated time spent carrying weight + doing heavy household work + dancing + exercise per week |
| InveCe.Ab | Frequency for a selection of activities was available (cycling, dancing, gymnastics, swimming, running, tennis, aerobics and walking). Activities were categorized according to the Compendium of physical activities^36^ into moderate intensity (metabolic equivalent (MET) ≥ 3.0) or vigorous intensity (MET ≥ 6.0). Weekly total frequency of the activities was calculated (vigorous activities counting double). Assuming 30 minutes spent on each activity per time, having a total weekly frequency of activities ≥ 5 (5 x 30 minutes = 150 minutes) was considered physically active. |
| ISA | International Physical Activity Questionnaire-based^37^ categories were dichotomized: low physical activity 🡪 not physically active, moderate and high physically active 🡪 physically active |
| KLOSCAD | Time in moderate intensity physical activity per week + time in vigorous physical activity x 2 |
| Leiden 85+ | / |
| LEILA 75+ | / |
| MAAS | Time spent on sports per week + time spent on light activities per week. If no data on sports or light exercise available: the average number of hours “up and about” per day was divided by tertiles. The highest tertile was considered physically active. |
| MYHAT | Categorical variable was available: no physical activity 🡪 not physically active, at least some physical activity 🡪 physically active |
| LRGS TUA | Categorical variable was available: answer to “Are you involved in exercises?” Not at all, very seldom, or seldom 🡪 not physically active; often, or very often 🡪 physically active |
| SALSA | Total MET-hour/week (based on 8 activities of moderate intensity), at least 7.5 MET hours/week was considered physically active (2.5 hours x 3 MET = 7.5 MET hours) |
| SAS | MET hours/week were available. At least 7.5 MET hours/week was considered physically active (2.5 hours x 3 MET = 7.5 MET hours) |
| SGS | Time spent on moderate-to-vigorous physical activity per day x 7 |
| SLAS II | Time in moderate intensity physical activity per week + time in vigorous physical activity x 2; if time spent on physical activity was missing: "often" engaging in physical exercises, walking, active sports or taiji was also considered physically active |
| MAS | Time spent on a selection of activities (bowling, golf, tennis, swimming, dancing, jogging, cycling, aerobics, other sports). Responses given as times (but not always clear what is intended). Activities were categorized according to the Compendium of physical activities^36^ into moderate intensity (metabolic equivalent (MET) ≥ 3.0) or vigorous intensity (MET ≥ 6.0). Time in moderate intensity physical activity per week + time in vigorous physical activity x 2 |
| ZARADEMP | / |

1. **Distribution of exposure to modifiable risk and protective factors included in the LIBRA score**

| Cohort | Physically inactive,  n (%) | Active smoker,  n (%) | Low-to-moderate alcohol consumption,  n (%) | Healthy diet, n (%) | Cognitively active,  n (%) | CHD,  n (%) | CKD,  n (%) | Hyper-tension,  n (%) | Dys-lipidemia,  n (%) | Diabetes, n (%) | Obesity,  n (%) | Depression, n (%) |
| --- | --- | --- | --- | --- | --- | --- | --- | --- | --- | --- | --- | --- |
| Bambui | 1148 (86) | 225 (17) | - | - | 162 (12) | 177 (13) | - | 828 (62) | 514 (39) | 197 (15) | 177 (13) | 493 (37) |
| CLAS | 627 (34) | 295 (16) | - | 1738 (94) | 511 (27) | 170 (15) | - | 1087 (58) | 724 (43) | 356 (18) | 50 (3) | 99 (5) |
| EAS | 914 (89) | 64 (6) | 54 (5) | - | 173 (27) | 256 (52) | - | 703 (69) | 203 (36) | 176 (17) | 233 (29) | 96 (9) |
| EPIDEMCA | 549 (80) | 92 (13) | 102 (15) | 198 (29) | 290 (42) | - | - | 467 (68) | 87 (15) | 80 (12) | 45 (7) | 274 (40) |
| ESPRIT | 290 (16) | 127 (6) | 934 (49) | - | 263 (14) | 235 (12) | - | 1262 (64) | 1657 (84) | 177 (9) | 169 (9) | 589 (30) |
| the H70 study | 102 (22) | 600 (77) | 89 (11) | - | 230 (27) | 148 (16) | - | 766 (85) | 758(84) | 127 (14) | 181 (21) | 134 (15) |
| HELIAD | 494 (49) | 106 (11) | 182 (18) | 261 (27) | 132 (13) | 95 (10) | 193 (19) | 707 (71) | 730 (73) | 220 (22) | 376 (38) | 178 (18) |
| InveCe.Ab | 829 (75) | 97 (9) | - | 688 (62) | 244 (22) | 112 (10) | 132 (12) | 650 (59) | 850 (78) | 250 (23) | 340 (35) | 171 (15) |
| ISA | 382 (31) | 504 (43) | - | 483 (41) | 1015 (84) | 169 (14) | - | 122 (10) | - | 26 (2) | 62 (6) | 415 (33) |
| KLOSCAD | 3738 (73) | 570 (11) | 720 (14) | - | - | 336 (7) | 16 (0.3) | 2628 (52) | 1204 (24) | 975 (19) | 163 (3) | 1007 (20) |
| Leiden 85+ | - | 77 (16) | 59 (12) | - | - | 117 (24) | 432 (90) | 453 (93) | 336 (70) | 209 (43) | 117 (24) | 75 (16) |
| LEILA 75+ | - | 289 (32) | 529 (59) | - | 184 (21) | 75 (8) | - | 729 (82) | - | 205 (23) | - | 327 (37) |
| LRGS TUA | 576 (58) | 165 (16) | 2 (0.2) | - | 324 (33) | 89 (9) | 17 (2) | 691 (69) | 667 (66) | 262 (26) | 120 (12) | - |
| MAAS | 1039 (70) | 454 (28) | 617 (39) | - | 527 (32) | 189 (12) | 68 (4) | 484 (29) | 162 (10) | 72 (4) | 294 (18) | 385 (24) |
| MYHAT | 242 (15) | 107 (6) | 123 (7) | - | 296 (30) | 239 (14) | - | 1357 (82) | 1242 (75) | 360 (22) | 513 (32) | 219 (13) |
| SALSA | 169 (12) | 163 (11) | 144 (10) | - | 387 (27) | 267 (18) | - | 989 (67) | 984 (68) | 466 (32) | 611 (43) | 424 (29) |
| SAS | 351 (21) | 169 (10) | - | - | 487 (31) | 193 (12) | 211 (17) | 895 (54) | 609 (37) | 221 (13) | 110 (7) | 244 (15) |
| SGS | 197 (24) | 85 (8) | - | - | 817 (78) | 132 (13) | 72 (7) | 736 (70) | 122 (12) | 130 (12) | 12 (1) | 10 (1) |
| SLAS II | 17 (1) | 120 (8) | 6 (0.4) | 60 (4) | 323 (23) | 117 (8) | 69 (5) | 822 (57) | 1078 (75) | 110 (8) | 96 (7) | 13 (1) |
| MAS | 396 (45) | 32 (4) | 314 (35) | 316 (35) | 242 (27) | 162 (18) | 307 (34) | 760 (84) | 681 (76) | 137 (15) | 206 (23) | 156 (17) |
| ZARADEMP | - | 452 (14) | 55 (2) | - | - | 218 (7) | - | 2188 (68) | 197 (6) | 393 (12) | 730 (23) | 535 (17) |

Abbreviations: interquartile range (IQR), LIfestyle for BRAin health index (LIBRA; theoretical range: -5.9 to +12.7), coronary heart disease (CHD), chronic kidney disease (CKD); (-): factor not available; missing data: Bambui: physical inactivity: n=2, CHD: n=9, dyslipidemia: n=1, diabetes: n=3, obese: n=15, depression: n=5; CLAS: physical inactivity: n=8; smoking: n=3, healthy diet: n=23, cognitive activity: n=5, CHD: n=766, dyslipidemia: n=182, diabetes: n=155, obese: n=34, depression: n=1; EAS: low-to-moderate alcohol consumption: n=2, cognitive activity: n=384, CHD: n=527, dyslipidemia: n=454, diabetes: n=1, obese: n=217; EPIDEMCA: physical inactivity: n=2186, smoking: n=2, healthy diet: n=3, hypertension: n=4, dyslipidemia: n=113, diabetes: n=10, obese: n=26; ESPRIT: physical inactivity: n=166, smoking: n=1, low-to-moderate alcohol consumption: n=65, cognitive activity: n=168, hypertension: n=2, obese: n=13, depression: n=9; the H70 study: physical inactivity: n=442, smoking: n=123, low-to-moderate alcohol consumption: n=65, cognitive activity: n=43, diabetes: n=1, obese: n=25, depression: n=14; HELIAD: smoking: n=9, low-to-moderate alcohol consumption: n=12, healthy diet: n=22, CHD: n=10, CKD: n=1, hypertension: n=9, dyslipidemia l: n=1, diabetes: n=2, obese: n=11; InveCe.Ab: physical inactivity: n=1, healthy diet: n=3, CHD: n=3, CKD: n=3, hypertension: n=3, dyslipidemia: n=11, obese: n=122, depression: n=3; ISA: smoking: n=78, healthy diet: n=72, cognitive activity: n=33, CHD: n=2, hypertension: n=13, diabetes: n=4, obese: n=195; KLOSCAD: physical inactivity: n=5, smoking: n=21, low-to-moderate alcohol consumption: n=17, CHD: n=10, hypertension: n=22, dyslipidemia: n=6, diabetes: n=6, obese: n=64, depression: n=15; Leiden 85+: low-to-moderate alcohol consumption: n=2, CHD: n=5, CKD: n=7, dyslipidemia: n=2, obese: n=7, depression: n=30; LRGS TUA: physical inactivity: n=15, low-to-moderate alcohol consumption: n=64, cognitive activity: n=12, obese: n=4; MAAS: physical inactivity: n=160, smoking: n=6, low-to-moderate alcohol consumption: n=53, cognitive activity: n=2, depression: n=10; MYHAT: smoking: n=4, low-to-moderate alcohol consumption: n=1, cognitive activity: n=657, dyslipidemia: n=2, obese: n=29; SALSA: physical inactivity: n=11, low-to-moderate alcohol consumption: n=2, cognitive activity: n=34, CHD: n=2, dyslipidemia: n=29, obese: n=65, depression: n=7; SAS: physical inactivity: n=9, smoking: n=2, cognitive activity: n=63, CHD: n=3, CKD: n=394, obese: n=4, depression: n=1; SGS: physical inactivity: n=213, smoking: n=5, obese: n=173; SLAS II: low-to-moderate alcohol consumption: n=11, healthy diet: n=8, CKD: n=88, diabetes: n=85, obese: n=33, depression: n=2; MAS: physical inactivity: n=17, smoking: n=6, low-to-moderate alcohol consumption: n=1, cognitive activity: n=19, CHD: n=6, dyslipidemia: n=1, obese: n=15, depression: n=1; ZARADEMP: low-to-moderate alcohol consumption: n=21, CHD: n=58, hypertension n=5, obese: n=6, diabetes: n=22, depression: n=131

References

1. Schiepers OJG, Kohler S, Deckers K, et al. Lifestyle for Brain Health (LIBRA): a new model for dementia prevention. *Int J Geriatr Psychiatry*. Jan 2018;33(1):167-175. doi:10.1002/gps.4700

2. Ferguson Bea. Estimating permanent income using indicator variables. In: Murray C, Evans D, eds. *Health Systems Performance Assessment: Debates, Methods and Empiricism*. World Health Organization; 2003.

3. Rose D, Harrison E. The european socio-economic classification: A new social class schema for comparative European research. *Eur Soc*. 2007;9(3):459-490. doi:10.1080/14616690701336518

4. Molloy DW, Alemayehu E, Roberts R. Reliability of a Standardized Mini-Mental State Examination compared with the traditional Mini-Mental State Examination. *Am J Psychiatry*. Jan 1991;148(1):102-5. doi:10.1176/ajp.148.1.102

5. Castro-Costa E, Fuzikawa C, Uchoa E, Firmo JO, Lima-Costa MF. Norms for the mini-mental state examination: adjustment of the cut-off point in population-based studies (evidences from the Bambui health aging study). *Arq Neuropsiquiatr*. Sep 2008;66(3A):524-8. doi:10.1590/s0004-282x2008000400016

6. Morris JC. The Clinical Dementia Rating (CDR): current version and scoring rules. *Neurology*. Nov 1993;43(11):2412-4. doi:10.1212/wnl.43.11.2412-a

7. American Psychiatric Association. Task Force on DSM-IV. *Diagnostic and statistical manual of mental disorders : DSM-IV*. 4th ed. American Psychiatric Association; 1994.

8. American Psychiatric Association. Work Group to Revise DSM-III. *Diagnostic and statistical manual of mental disorders : DSM-III-R*. 3rd ed. American Psychiatric Association; 1987.

9. Knopman DS, Ryberg S. A verbal memory test with high predictive accuracy for dementia of the Alzheimer type. *Arch Neurol*. Feb 1989;46(2):141-5. doi:10.1001/archneur.1989.00520380041011

10. Hendrie HC, Lane KA, Ogunniyi A, et al. The development of a semi-structured home interview (CHIF) to directly assess function in cognitively impaired elderly people in two cultures. *Int Psychogeriatr*. Dec 2006;18(4):653-66. doi:10.1017/S104161020500308X

11. Teng EL, Chui HC. The Modified Mini-Mental State (3MS) examination. *J Clin Psychiatry*. Aug 1987;48(8):314-8.

12. Gonzalez HM, Mungas D, Reed BR, Marshall S, Haan MN. A new verbal learning and memory test for English- and Spanish-speaking older people. *J Int Neuropsychol Soc*. Jul 2001;7(5):544-55. doi:10.1017/s1355617701755026

13. Mungas D, Reed BR, Marshall SC, Gonzalez HM. Development of psychometrically matched English and Spanish language neuropsychological tests for older persons. *Neuropsychology*. Apr 2000;14(2):209-23. doi:10.1037//0894-4105.14.2.209

14. Jorm AF, Jacomb PA. The Informant Questionnaire on Cognitive Decline in the Elderly (IQCODE): socio-demographic correlates, reliability, validity and some norms. *Psychol Med*. Nov 1989;19(4):1015-22. doi:10.1017/s0033291700005742

15. Weir CB, Jan A. BMI Classification Percentile And Cut Off Points. *StatPearls*. 2022.

16. Kromhout D, Spaaij CJK, de Goede J, et al. The 2015 Dutch food-based dietary guidelines. *Eur J Clin Nutr*. Aug 2016;70(8):869-878. doi:10.1038/ejcn.2016.52

17. Levey AS, Stevens LA, Schmid CH, et al. A new equation to estimate glomerular filtration rate. *Ann Intern Med*. May 5 2009;150(9):604-12. doi:10.7326/0003-4819-150-9-200905050-00006

18. Levey AS, Coresh J, Greene T, et al. Using standardized serum creatinine values in the modification of diet in renal disease study equation for estimating glomerular filtration rate. *Ann Intern Med*. Aug 15 2006;145(4):247-54. doi:10.7326/0003-4819-145-4-200608150-00004

19. Xiao J, Huang B, Shen H, et al. Association between social support and health-related quality of life among Chinese seafarers: A cross-sectional study. *PLoS One*. 2017;12(11):e0187275. doi:10.1371/journal.pone.0187275

20. Schinka JA, McBride A, Vanderploeg RD, Tennyson K, Borenstein AR, Mortimer JA. Florida Cognitive Activities Scale: initial development and validation. *J Int Neuropsychol Soc*. Jan 2005;11(1):108-16. doi:10.1017/S1355617705050125

21. Wu W, Zhao Q, Xiao Z, Liang X, Luo J, Ding D. Association of combined engagement in cognitive and physical activity with domain-specific cognitive function: The Shanghai Aging Study. *Int J Geriatr Psychiatry*. Jan 2021;36(1):116-126. doi:10.1002/gps.5403

22. Launer LJ, Brayne C, Dartigues JF, Hofman A. European Studies on the Incidence of Dementing Diseases *Neuroepidemiology*. 1992;11doi:10.1159/000111006

23. Sanchez-Lopez Mdel P, Dresch V. The 12-Item General Health Questionnaire (GHQ-12): reliability, external validity and factor structure in the Spanish population. *Psicothema*. Nov 2008;20(4):839-43.

24. Yesavage JA, Brink TL, Rose TL, et al. Development and validation of a geriatric depression screening scale: a preliminary report. *J Psychiatr Res*. 1982;17(1):37-49. doi:10.1016/0022-3956(82)90033-4

25. Sheikh JI, Yesavage JA. Geriatric depression scale (GDS); recent evidence and development of a shorter version. *Gerontologist*. 1986;(5):165-173.

26. Copeland JR, Dewey ME, Griffiths-Jones HM. A computerized psychiatric diagnostic system and case nomenclature for elderly subjects: GMS and AGECAT. *Psychol Med*. Feb 1986;16(1):89-99. doi:10.1017/s0033291700057779

27. Lecrubier Y, Sheehan DV, Weiller E, et al. The Mini International Neuropsychiatric Interview (MINI). A short diagnostic structured interview: Reliability and validity according to the CIDI. *Eur Psychiat*. 1997;12(5):224-231. doi:Doi 10.1016/S0924-9338(97)83296-8

28. Radloff LS. The CES-D Scale: A self-report depression scale for research in the general population. *Applied Psychological Measurement*. 1977;1(3):385-401.

29. Montgomery SA, Asberg M. A new depression scale designed to be sensitive to change. *Br J Psychiatry*. Apr 1979;134:382-9. doi:10.1192/bjp.134.4.382

30. Whooley MA, Avins AL, Miranda J, Browner WS. Case-finding instruments for depression. Two questions are as good as many. *J Gen Intern Med*. Jul 1997;12(7):439-45. doi:10.1046/j.1525-1497.1997.00076.x

31. Bae JN, Cho MJ. Development of the Korean version of the Geriatric Depression Scale and its short form among elderly psychiatric patients. *J Psychosom Res*. Sep 2004;57(3):297-305. doi:10.1016/j.jpsychores.2004.01.004

32. Arrindell WA, Ettema JHM. *SCL-90: A Multidimensional Indicator of Psychopathology*. Swets & Zeitlinger; 1986.

33. World Health Organisation. *Global action plan for the prevention and control of NCDs 2013–2020*. 2013.

34. National Cholesterol Education Program. Second Report of the Expert Panel on Detection, Evaluation, and Treatment of High Blood Cholesterol in Adults (Adult Treatment Panel II). *Circulation*. Mar 1994;89(3):1333-445. doi:10.1161/01.cir.89.3.1333

35. World Health Organization (WHO). *WHO Guidelines on Physical Activity and Sedentary Behaviour*. 2020.

36. Ainsworth BE, Haskell WL, Herrmann SD, et al. 2011 Compendium of Physical Activities: a second update of codes and MET values. *Med Sci Sports Exerc*. Aug 2011;43(8):1575-81. doi:10.1249/MSS.0b013e31821ece12

37. Craig CL, Marshall AL, Sjostrom M, et al. International physical activity questionnaire: 12-country reliability and validity. *Med Sci Sport Exer*. Aug 2003;35(8):1381-1395. doi:10.1249/01.Mss.0000078924.61453.Fb
