# Supplementary material for: Lifestyle and incident dementia: A COSMIC individual participant data meta‐analysis
Source: Alzheimers Dement. 2024 Apr 27;20(6):3972–86. [Article in Italian] doi: 10.1002/alz.13846 (PMC11180928; doi:10.1002/alz.13846)
Supplement: Supplementary file 3 — Supporting Information [file ALZ-20-3972-s004.docx]

**Supplementary material 3: Comparison of the analytical sample and excluded individuals**

Table 1: Demographic characteristics of the analytical sample and excluded individuals

| Cohort | Age, mean (SD) | | | Female sex, n (%) | | | Years of formal education, mean (SD) | | |
| --- | --- | --- | --- | --- | --- | --- | --- | --- | --- |
|  | Included | Excluded | P value | Included | Excluded | P value | Included | Excluded | P value |
| Bambui | 69 (7) | 73 (9) | **<.001** | 829 (62.1) | 135 (50.0) | **<.001** | 2.9 (3.0) | 1.8 (2.5) | **<.001** |
| CLAS | 71 (8) | 72 (8) | .462 | 1008 (53.9) | 757 (56.0) | .249 | 8.0 (5.1) | 8.3 (5.7) | .095 |
| EAS | 78 (5) | 79 (5) | **<.001** | 635 (62.1) | 783 (62.0) | .927 | 14.0 (3.5) | 12.4 (3.7) | **<.001** |
| EPIDEMCA | 74 (7) | 74 (7) | .089 | 407 (59.1) | 226 (66.5) | **.022** | 2.4 (4.3) | 1.5 (3.4) | **<.001** |
| ESPRIT | 73 (5) | 76 (7) | **<.001** | 1168 (58.9) | 145 (52.5) | **.045** | 10.3 (3.7) | 8.9 (4.0) | **<.001** |
| The H70 study | 74 (5) | 78 (6) | **<.001** | 672 (74.5) | 285 (89.4) | **<.001** | 9.6 (4.1) | 8.5 (3.2) | **<.001** |
| HELIAD | 73 (5) | 74 (6) | **<.001** | 597 (59.6) | 636 (59.9) | .888 | 7.8 (4.7) | 8.3 (5.3) | **.025** |
| InveCe.Ab | 72 (1) | 72 (1) | **<.001** | 598 (54.0) | 116 (54.2) | .960 | 6.9 (3.4) | 6.1 (3.3) | **.003** |
| ISA | 74 (8) | 76 (10) | **<.001** | 639 (51.4) | 509 (56.2) | **.025** | 3.5 (4.6) | 3.6 (4.6) | .629 |
| KLOSCAD | 70 (6) | 73 (8) | **<.001** | 2898 (56.7) | 1015 (59.7) | **.031** | 8.4 (5.3) | 6.2 (5.3) | **<.001** |
| Leiden 85+ | *85* | *85* | - | 315 (65.0) | 82 (71.9) | .156 | 7.5 (3.0) | 7.0 (2.4) | .054 |
| LEILA 75+ | 81 (5) | 85 (5) | **<.001** | 653 (73.3) | 311 (83.2) | **<.001** | 11.9 (1.8) | 11.1 (1.2) | **<.001** |
| LRGS TUA | 68 (5) | 70 (7) | **<.001** | 525 (52.2) | 683 (51.9) | .891 | 5.5 (4.1) | 4.8 (3.9) | **<.001** |
| MAAS | 52 (16) | 46 (17) | **<.001** | 796 (48.5) | 114 (63.3) | **<.001** | 11.2 (3.4) | 11.7 (4.2) | .169 |
| MYHAT | 77 (7) | 79 (8) | **<.001** | 1028 (62.2) | 142 (53.4) | **.006** | 12.9 (2.4) | 12.6 (2.3) | .052 |
| SALSA | 70 (7) | 73 (9) | **<.001** | 856 (58.2) | 188 (59.1) | .761 | 7.6 (5.4) | 5.4 (4.7) | **<.001** |
| SAS | 72 (7) | 70 (10) | **<.001** | 900 (54.3) | 1235 (56.7) | .145 | 11.9 (4.0) | 11.3 (4.3) | **<.001** |
| SGS | 73 (6) | 74 (6) | **<.001** | 583 (55.5) | 899 (56.9) | .475 | 11.4 (2.6) | 10.9 (2.5) | **<.001** |
| SLAS II | 66 (7) | 68 (9) | **<.001** | 929 (64.8) | 1115 (60.7) | **.015** | 6.5 (4.3) | 5.0 (4.0) | **<.001** |
| MAS | 79 (5) | 81 (5) | **<.001** | 488 (54.2) | 84 (61.3) | .120 | 11.7 (3.5) | 11.1 (3.4) | .079 |
| ZARADEMP | 71 (9) | 78 (10) | **<.001** | 1784 (55.3) | 987 (62.6) | **<.001** | 7.6 (3.9) | 6.0 (3.6) | **<.001** |

NOTE. Comparisons done using Chi^2^ test and unpaired t-test, all participants were 85 years old in the Leiden 85+ study. Abbreviations: standard deviation (SD).

Table 2 (part 1): Prevalence of conditions that are part of the LIfestyle for BRAin health (LIBRA) index, in the analytical sample and excluded individuals

| Cohort | Ischemic heart disease, n (%) | | | Hypertension, n (%) | | | Dyslipidemia, n (%) | | |
| --- | --- | --- | --- | --- | --- | --- | --- | --- | --- |
|  | Included | Excluded | P value | Included | Excluded | P value | Included | Excluded | P value |
| Bambui | 177 (13.3) | 21 (13.6) | .942 | 828 (62.0) | 90 (56.0) | .221 | 514 (38.5) | 55 (34.4) | .310 |
| CLAS | 170 (15.4) | 129 (15.4) | .989 | 1087 (58.1) | 770 (58.7) | .707 | 724 (42.8) | 441 (38.2) | **.013** |
| EAS | 256 (51.7) | 213 (53.0) | .705 | 703 (68.8) | 699 (58.4) | **<.001** | 203 (35.7) | 54 (32.3) | .417 |
| EPIDEMCA | - | - | - | 467 (68.2) | 229 (68.2) | .995 | 87 (15.1) | 27 (10.4) | .068 |
| ESPRIT | 235 (11.9) | 50 (18.1) | **.003** | 1262 (63.7) | 180 (65.2) | .624 | 1657 (83.6) | 212 (76.8) | **.005** |
| The H70 study | 148 (16.4) | 13 (10.2) | .073 | 766 (84.9) | 102 (76.1) | **.010** | 758 (84.0) | 83 (55.7) | **<.001** |
| HELIAD | 95 (9.6) | 125 (12.4) | **.047** | 707 (71.3) | 708 (69.5) | .379 | 730 (73.0) | 674 (64.8) | **<.001** |
| InveCe.Ab | 112 (10.1) | 35 (17.1) | **.004** | 650 (58.9) | 111 (54.2) | .207 | 850 (77.6) | 154 (72.6) | .121 |
| ISA | 169 (13.6) | 108 (12.0) | .261 | 122 (9.9) | 94 (10.5) | .674 | - | - | - |
| KLOSCAD | 336 (6.6) | 84 (5.8) | .265 | 2628 (51.7) | 768 (55.1) | **.023** | 1204 (23.6) | 252 (17.3) | **<.001** |
| Leiden 85+ | 117 (24.4) | 22 (20.6) | .401 | 453 (93.4) | 89 (79.5) | **<.001** | 336 (69.6) | 50 (64.1) | .334 |
| LEILA 75+ | 75 (8.4) | 23 (6.5) | .267 | 729 (81.8) | 218 (71.2) | **<.001** | - | - | - |
| LRGS TUA | 89 (8.9) | 150 (11.4) | **.045** | 691 (68.7) | 974 (74.0) | **.005** | 667 (66.3) | 802 (60.9) | **.008** |
| MAAS | 189 (11.5) | 36 (3.5) | **<.001** | 484 (29.5) | 126 (48.8) | **<.001** | 162 (9.9) | 33 (3.3) | **<.001** |
| MYHAT | 239 (14.5) | 45 (17.1) | .261 | 1357 (82.1) | 220 (83.0) | .714 | 1242 (75.2) | 171 (65.0) | **<.001** |
| SALSA | 267 (18.2) | 63 (20.7) | .311 | 989 (67.2) | 219 (69.5) | .430 | 984 (68.2) | 137 (60.4) | **.019** |
| SAS | 193 (11.7) | 281 (13.0) | .208 | 895 (54.0) | 1125 (52.1) | .231 | 609 (36.8) | 681 (31.6) | **.001** |
| SGS | 132 (12.6) | 208 (13.3) | .566 | 736 (70.1) | 1059 (67.5) | .160 | 122 (11.6) | 135 (8.7) | **.013** |
| SLAS II | 117 (8.2) | 187 (10.2) | **.049** | 822 (57.4) | 1183 (64.4) | **<.001** | 1078 (75.2) | 1384 (75.3) | .941 |
| MAS | 162 (18.1) | 36 (26.5) | **.021** | 760 (84.4) | 109 (79.6) | .148 | 681 (75.8) | 97 (71.3) | .265 |
| ZARADEMP | 218 (6.9) | 117 (7.7) | .327 | 2188 (67.9) | 1051 (67.9) | .571 | 197 (6.1) | 60 (3.8) | **.001** |

NOTE. Comparisons done using Chi^2^ test and unpaired t-test. Abbreviations: standard deviation (SD).

Table 2 (part 2): Prevalence of conditions that are part of the LIfestyle for BRAin health (LIBRA) index, in the analytical sample and excluded individuals

| Cohort | Obesity, n (%) | | | Diabetes, n (%) | | | Depression, n (%) | | |
| --- | --- | --- | --- | --- | --- | --- | --- | --- | --- |
|  | Included | Excluded | P value | Included | Excluded | P value | Included | Excluded | P value |
| Bambui | 177 (13.4) | 12 (9.2) | .178 | 197 (14.8) | 16 (18.2) | .386 | 493 (37.0) | 89 (49.7) | **.001** |
| CLAS | 50 (2.7) | 50 (3.9) | .058 | 306 (17.7) | 285 (23.7) | **<.001** | 99 (5.3) | 63 (4.8) | .493 |
| EAS | 233 (28.9) | 97 (31.6) | .387 | 176 (17.2) | 218 (18.2) | .538 | 96 (9.4) | 143 (15.7) | **<.001** |
| EPIDEMCA | 45 (6.8) | 18 (6.0) | .665 | 80 (11.8) | 36 (10.9) | .683 | 274 (39.8) | 163 (47.9) | **.013** |
| ESPRIT | 169 (8.6) | 28 (10.4) | .330 | 177 (8.9) | 40 (14.5) | **.003** | 589 (29.8) | 102 (37.8) | **.008** |
| The H70 study | 181 (20.6) | 15 (21.7) | .828 | 127 (14.1) | 21 (20.6) | .080 | 134 (15.1) | 35 (34.3) | **<.001** |
| HELIAD | 376 (38.0) | 338 (33.8) | .054 | 220 (22.0) | 247 (23.8) | .347 | 178 (17.8) | 258 (25.3) | **<.001** |
| InveCe.Ab | 340 (34.5) | 57 (36.1) | .703 | 250 (22.6) | 49 (22.9) | .920 | 171 (15.5) | 54 (26.6) | **<.001** |
| ISA | 62 (5.9) | 5 (5.1) | .727 | 26 (2.1) | 20 (2.2) | .855 | 415 (33.4) | 327 (36.1) | .182 |
| KLOSCAD | 163 (3.2) | 47 (3.6) | .550 | 975 (19.1) | 333 (22.8) | **.002** | 1007 (19.8) | 371 (26.7) | **<.001** |
| Leiden 85+ | 117 (24.5) | 18 (26.9) | .671 | 209 (43.1) | 30 (27.3) | **.002** | 75 (16.5) | 10 (18.2) | .750 |
| LEILA 75+ | - | - | - | 205 (23.0) | 81 (22.5) | .846 | 327 (36.7) | 67 (17.9) | **<.001** |
| LRGS TUA | 120 (12.0) | 175 (13.4) | .327 | 262 (26.0) | 436 (33.1) | **<.001** | - | - | - |
| MAAS | 294 (17.9) | 35 (19.4) | .608 | 72 (4.4) | 35 (3.4) | .232 | 385 (23.6) | 18 (18.2) | .217 |
| MYHAT | 513 (31.6) | 57 (22.8) | **.005** | 360 (21.8) | 61 (23.1) | .629 | 219 (13.3) | 43 (16.3) | .182 |
| SALSA | 611 (43.5) | 75 (34.1) | **.009** | 466 (31.7) | 125 (40.3) | **.003** | 424 (29.0) | 97 (36.6) | **.013** |
| SAS | 110 (6.7) | 98 (4.5) | **.004** | 221 (13.3) | 303 (14.0) | .535 | 244 (14.7) | 428 (19.9) | **<.001** |
| SGS | 12 (1.4) | 40 (3.**7)** | **.001** | 130 (12.4) | 216 (13.9) | .276 | 10 (1.0) | 12 (0.8) | .617 |
| SLAS II | 96 (6.9) | 141 (8.4) | .104 | 110 (8.2) | 206 (13.3) | **<.001** | 13 (0.9) | 57 (3.1) | **<.001** |
| MAS | 206 (23.3) | 30 (24.0) | .858 | 137 (15.2) | 23 (16.8) | .636 | 156 (17.4) | 31 (23.0) | .114 |
| ZARADEMP | 730 (22.7) | 385 (24.9) | .095 | 393 (12.3) | 204 (13.1) | .396 | 535 (17.3) | 290 (26.2) | **<.001** |

NOTE. Comparisons done using Chi^2^ test and unpaired t-test. Abbreviations: standard deviation (SD).
