# Supplementary material for: Lifestyle and incident dementia: A COSMIC individual participant data meta‐analysis
Source: Alzheimers Dement. 2024 Apr 27;20(6):3972–86. [Article in Italian] doi: 10.1002/alz.13846 (PMC11180928; doi:10.1002/alz.13846)
Supplement: Supplementary file 4 — Supporting Information [file ALZ-20-3972-s007.docx]

**Supplementary material 4: LIfestyle for BRAin health (LIBRA) index across sociodemographic strata**


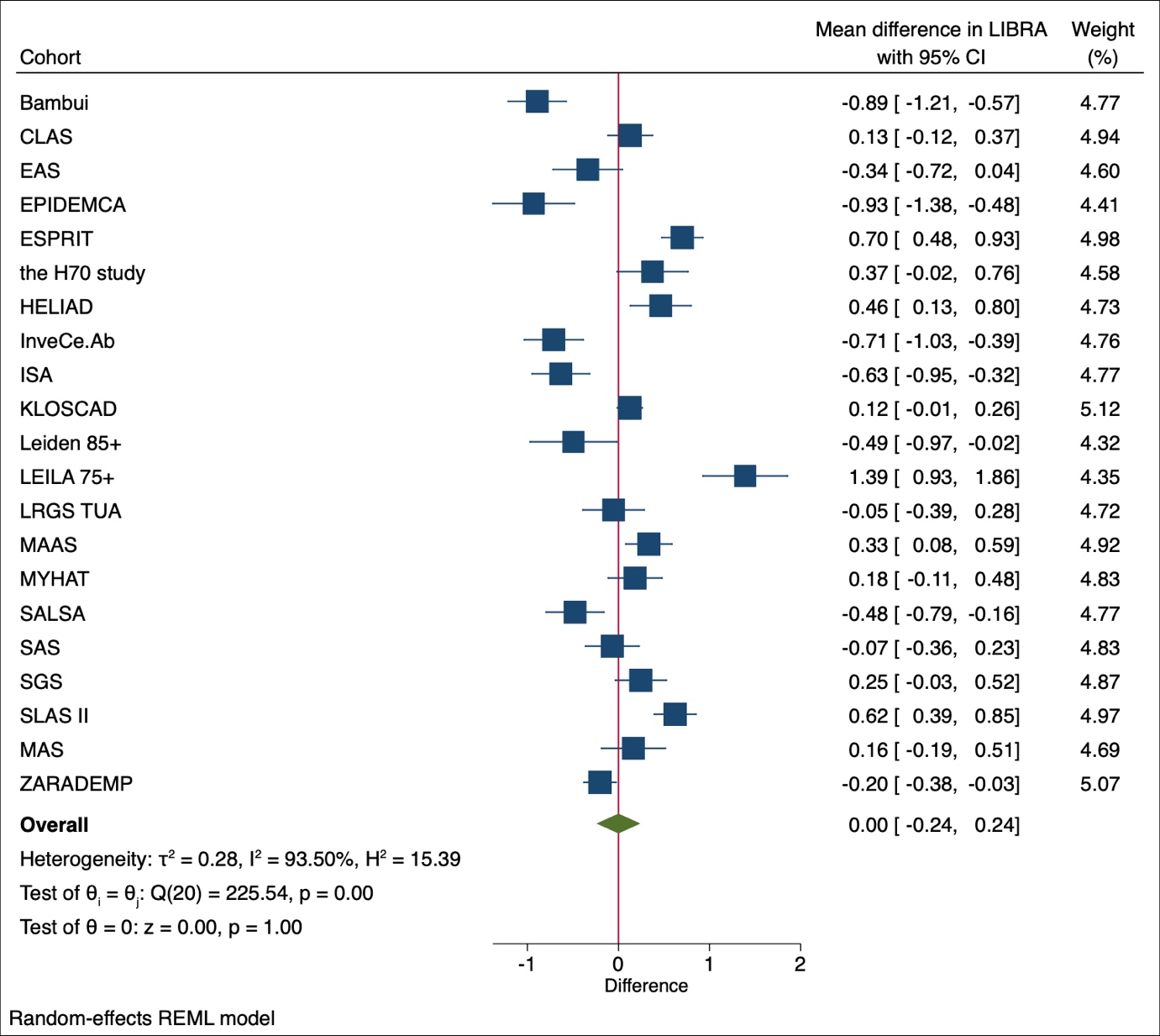


**Figure 1:** Pooled mean difference in LIBRA score between males and females was not significantly different from zero.

NOTE. Cohort-specific differences in mean LIBRA score between males and females were calculated (LIBRA_male_ – LIBRA_female_). These differences were pooled across cohorts. Abbreviations: LIfestyle for BRAin health (LIBRA), 95% confidence interval (95%CI)


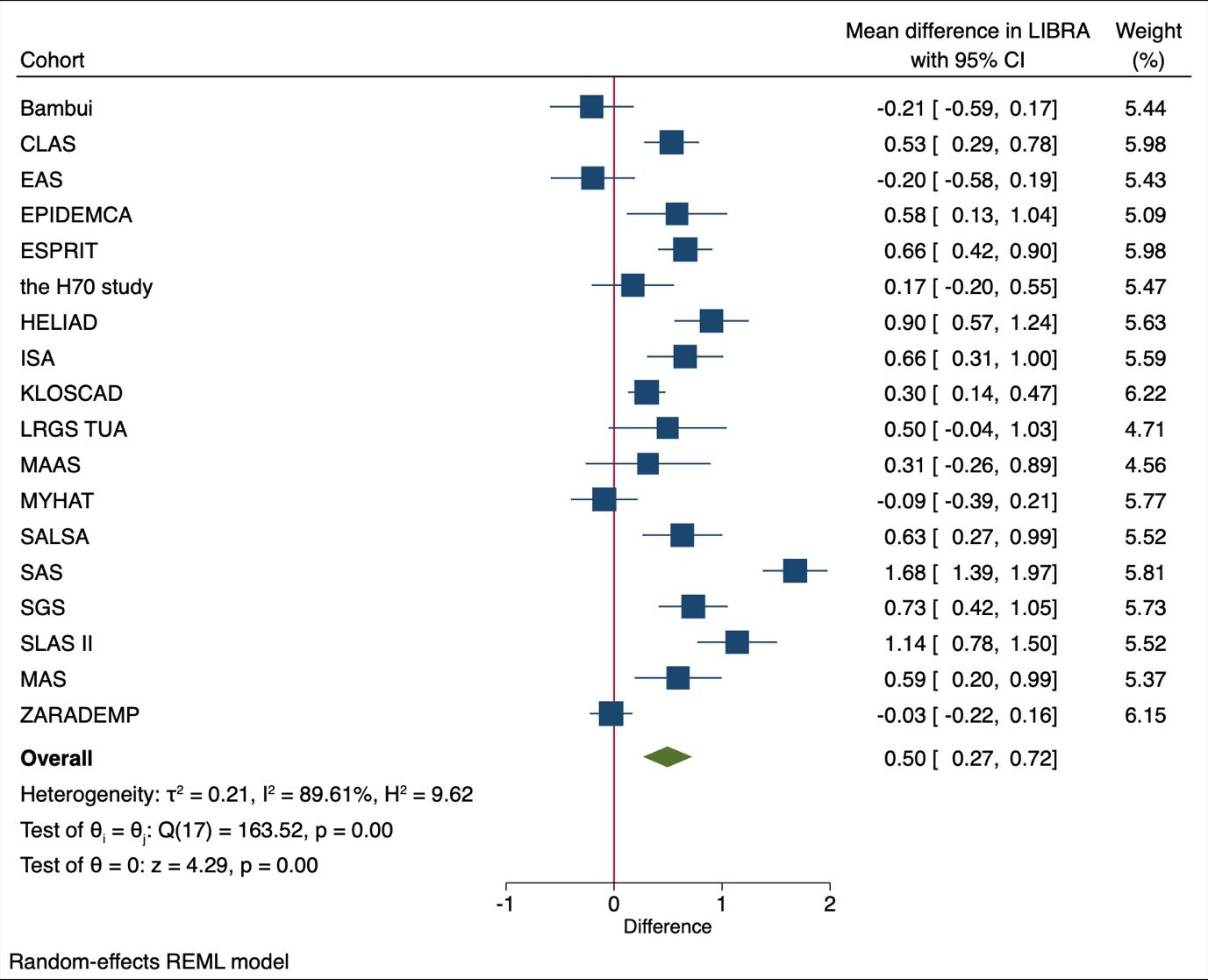


**Figure 2**: The LIBRA index was significantly smaller for people up to 75 years old compared to those older.

NOTE. Cohort-specific differences in mean LIBRA score between people up to 75 years old and people of 76 years old or more were calculated (LIBRA_≥76 years_ - LIBRA_≤75 years_). These differences were pooled across cohorts. Abbreviations: LIfestyle for BRAin health (LIBRA), 95% confidence interval (95%CI).


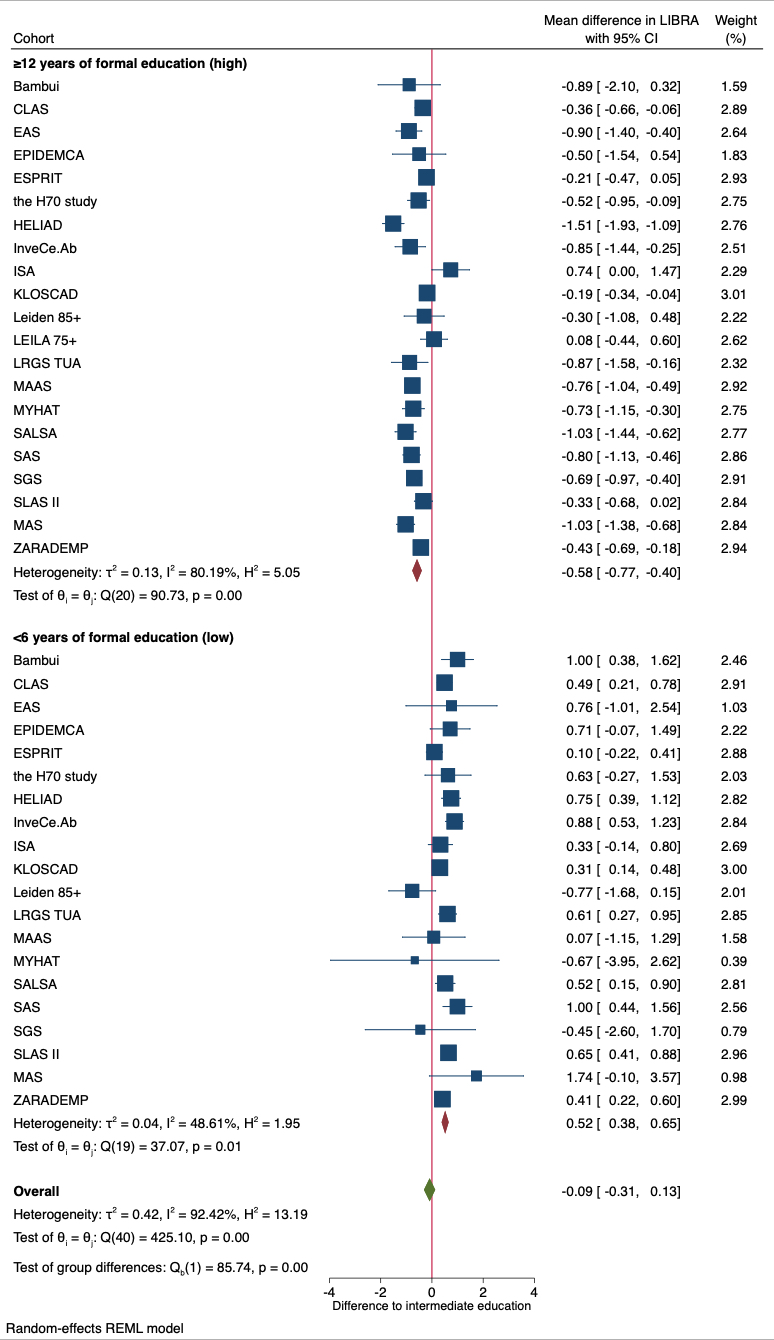


**Figure 3**: Compared to persons with an intermediate level of education, highly educated individuals had a lower LIBRA score whereas low educated individuals had a higher LIBRA score.

NOTE. Cohort-specific differences in mean LIBRA score between people with an intermediate level of education (6-11 years of formal education) and (1) a low level of education (<6 years of formal education) or (2) a high level of education (≥12 years of formal education), were calculated (LIBRA_high or low education_ – LIBRA_intermediate education_) and pooled across cohorts. Abbreviations: LIfestyle for BRAin health (LIBRA), 95% confidence interval (95%CI)

**
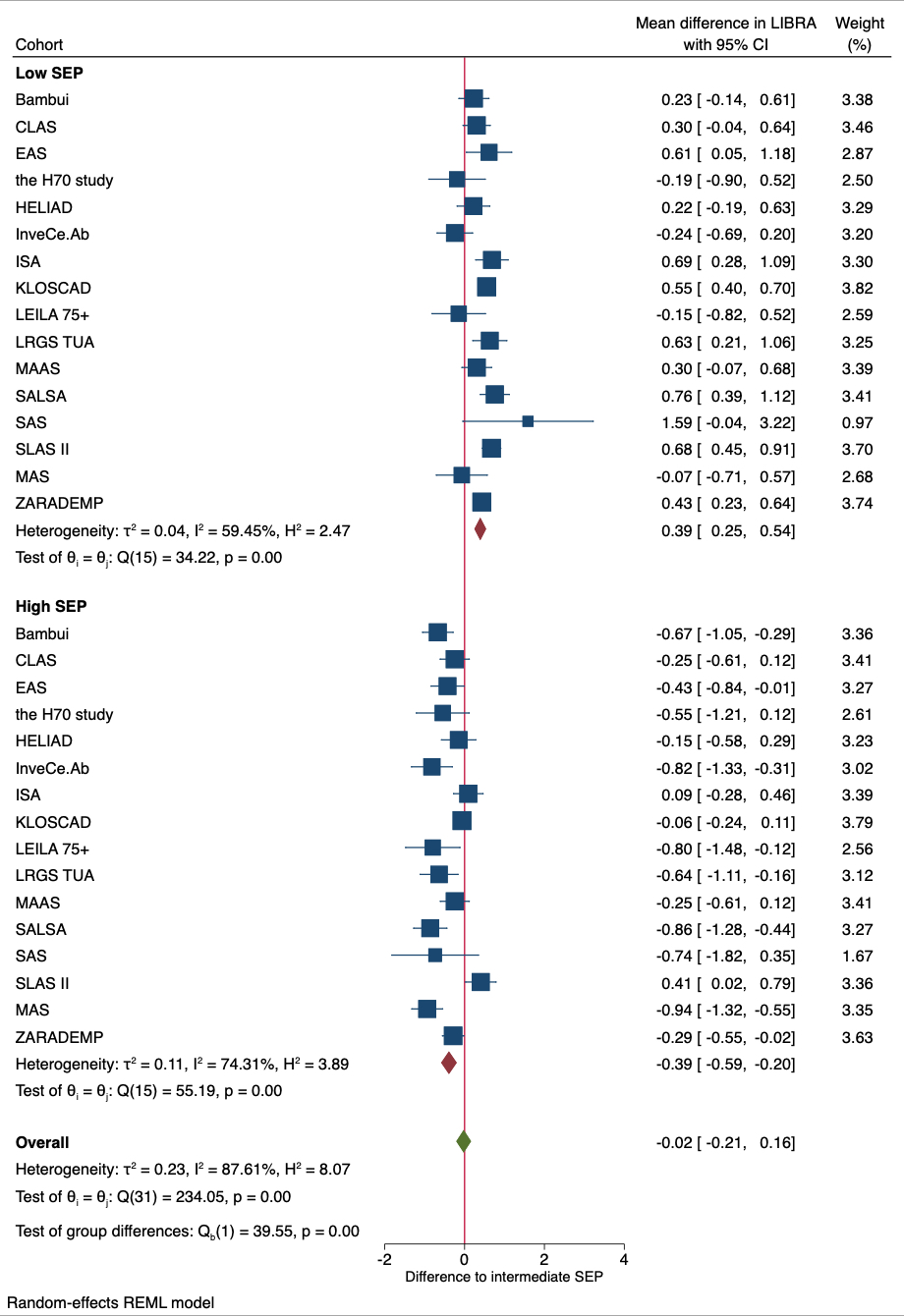
**

**Figure 4**: Compared to persons with an intermediate socioeconomic position (SEP), persons with a high SEP had a lower LIBRA score whereas persons with a low SEP had a higher LIBRA score.

NOTE. Cohort-specific differences in mean LIBRA score between people with an intermediate SEP and (1) a low SEP or (2) a high SEP, were calculated (LIBRA_high or low SEP_ – LIBRA_intermediate SEP_) and pooled across cohorts. Abbreviations: LIfestyle for BRAin health (LIBRA), 95% confidence interval (95%CI)
