# Supplementary material for: Lifestyle and incident dementia: A COSMIC individual participant data meta‐analysis
Source: Alzheimers Dement. 2024 Apr 27;20(6):3972–86. [Article in Italian] doi: 10.1002/alz.13846 (PMC11180928; doi:10.1002/alz.13846)
Supplement: Supplementary file 5 — Supporting Information [file ALZ-20-3972-s008.docx]

**Supplemental material 5: LIfesyle for BRAin health (LIBRA) score and dementia incidence: meta-analysis by sociodemographic characteristics and geographic location**


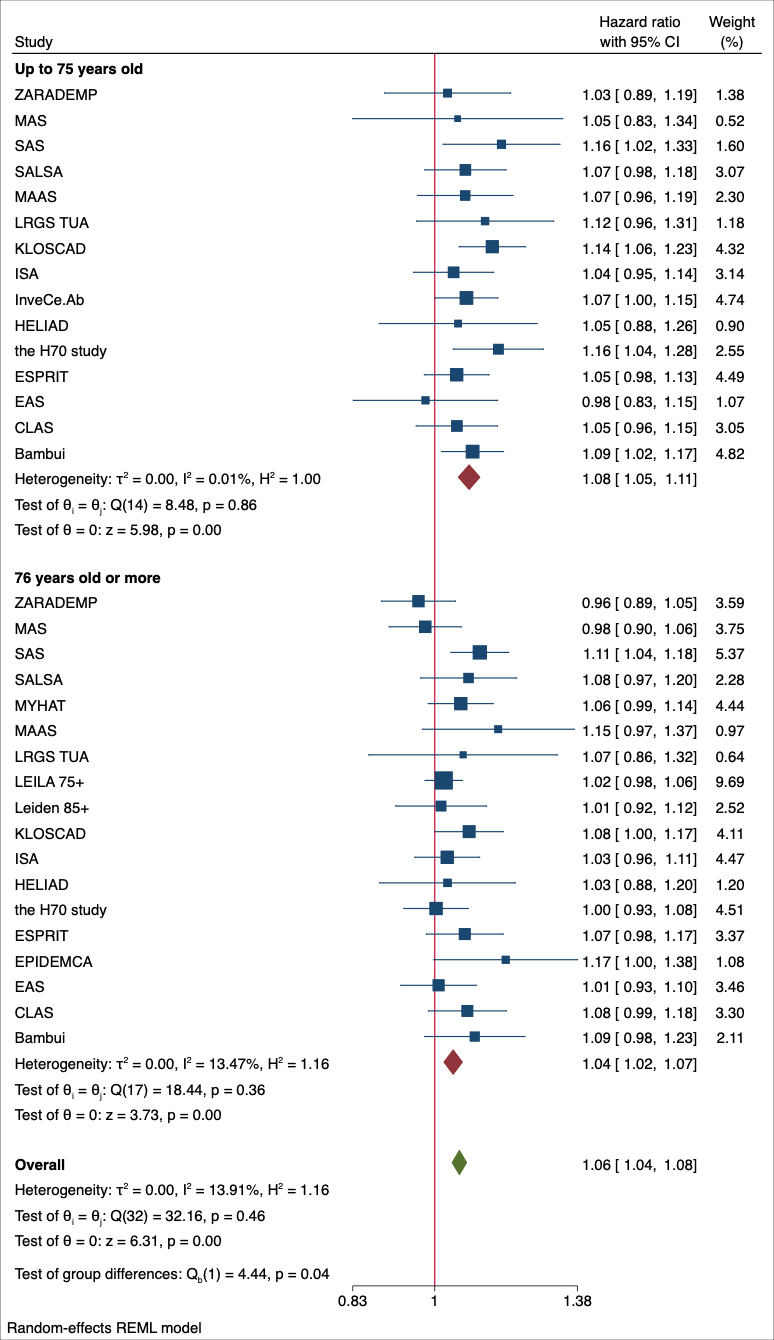


**Figure 1**: Pooled hazard ratio of a one-point increase in LIfestyle for BRAin health (LIBRA) score for incident dementia by baseline age

NOTE. Model 2, age on time axis, controlled for years of formal education and sex.


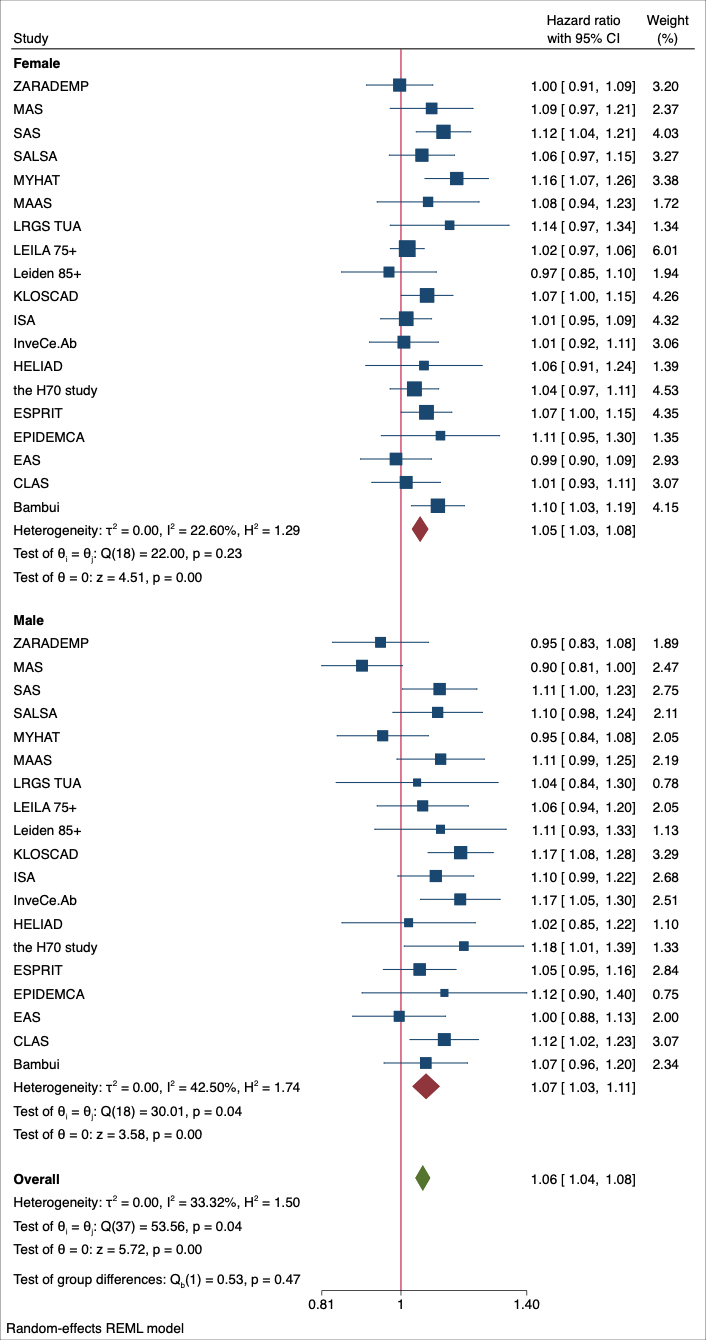


**Figure 2**: Pooled hazard ratio of a one-point increase in LIfestyle for BRAin health (LIBRA) score for incident dementia by sex

NOTE. Model 2, age on time axis, controlled for years of formal education.


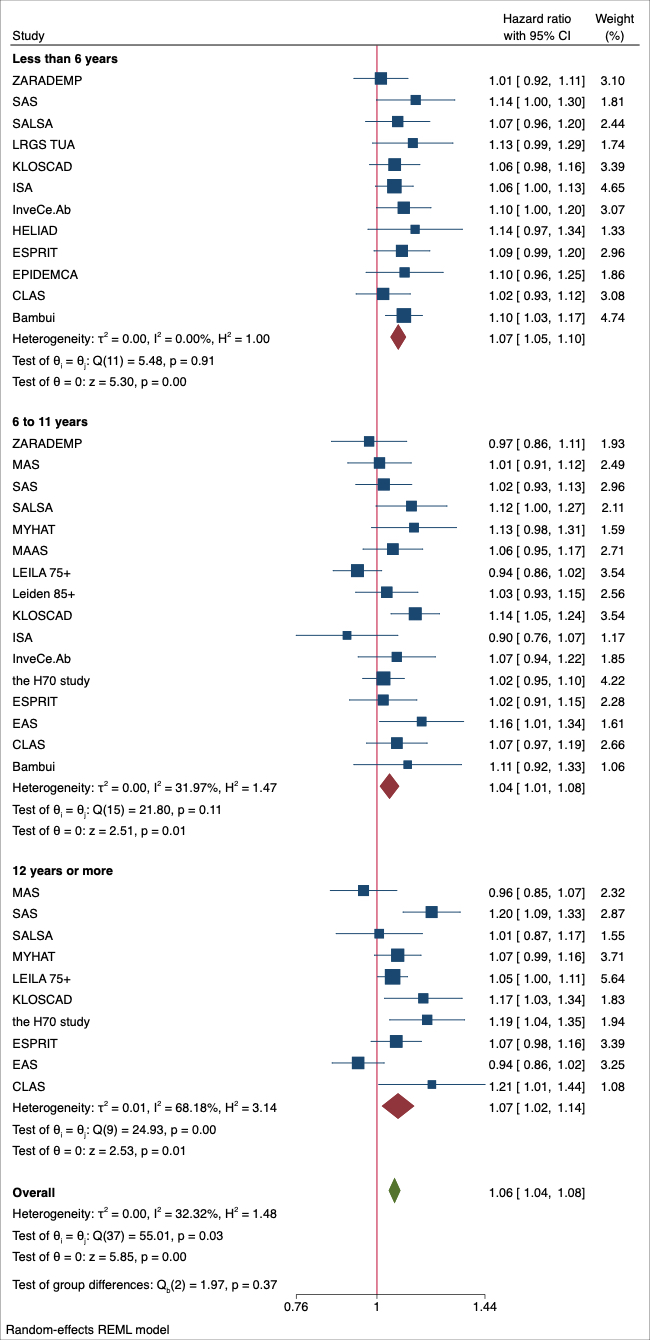


**Figure 3**: Pooled hazard ratio of a one-point increase in LIfestyle for BRAin health (LIBRA) score for incident dementia by years of formal education

NOTE. Model 2, age on time axis, controlled for sex.


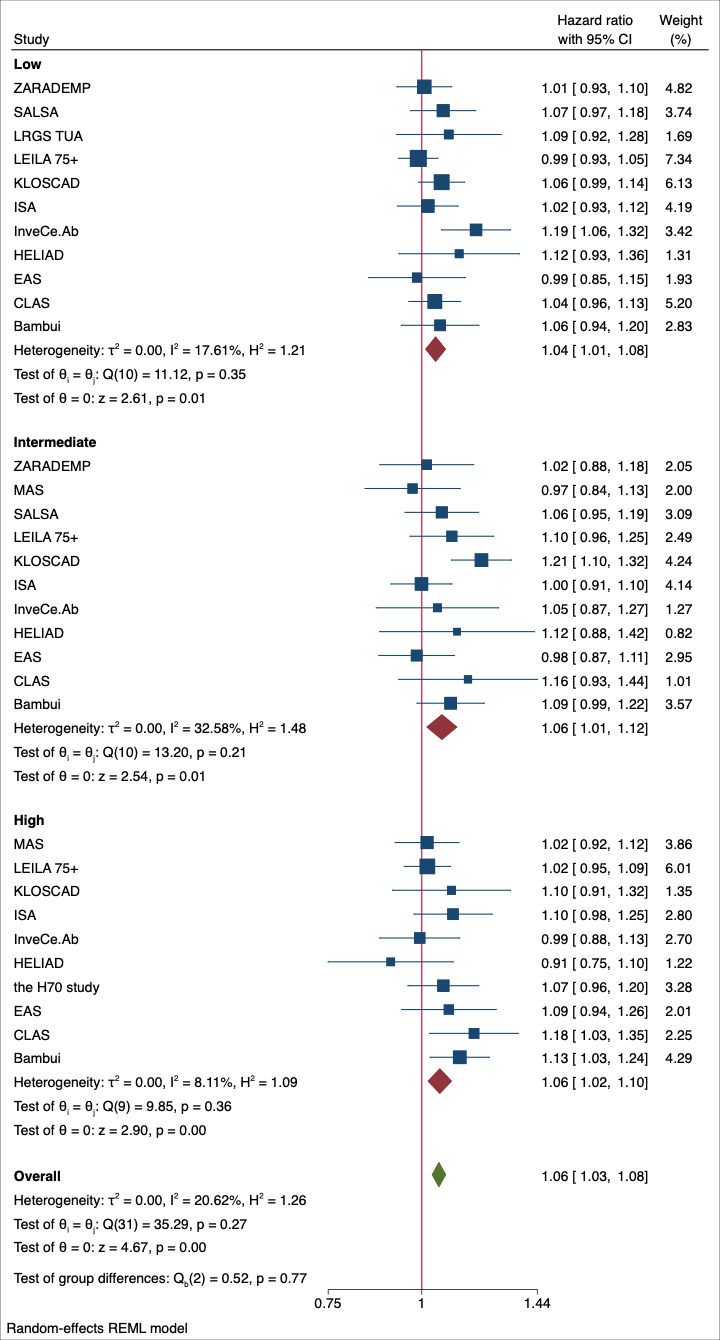


**Figure 4**: Pooled hazard ratio of a one-point increase in LIfestyle for BRAin health (LIBRA) score for incident dementia by socioeconomic position (SEP)

NOTE. Model 2, age on time axis, controlled for years of formal education and sex.


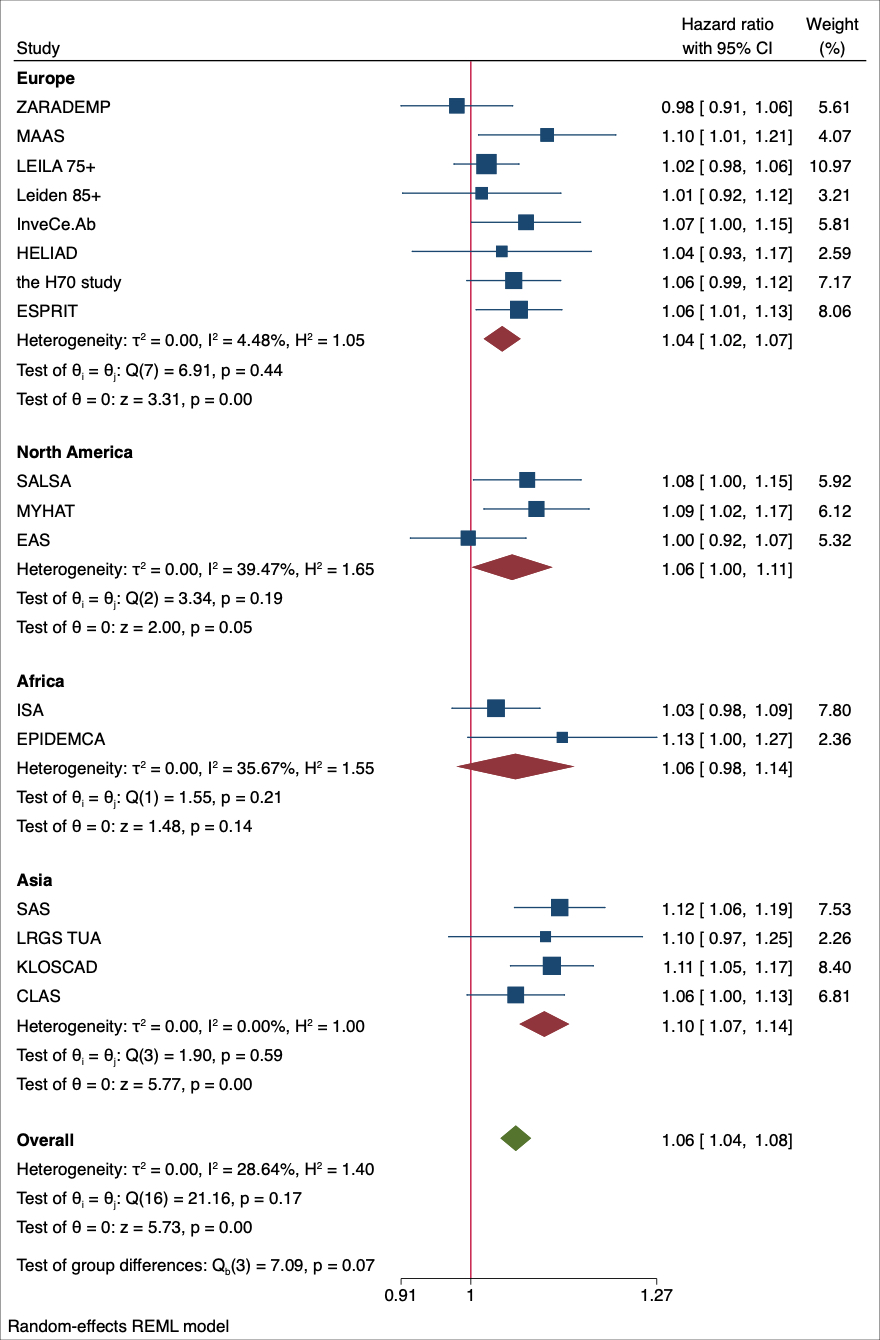


**Figure 5**: Pooled hazard ratio of a one-point increase in LIfestyle for BRAin health (LIBRA) score for incident dementia by continent

NOTE. Model 2, age on time axis, controlled for years of formal education and sex.
