# Supplementary material for: Lifestyle and incident dementia: A COSMIC individual participant data meta‐analysis
Source: Alzheimers Dement. 2024 Apr 27;20(6):3972–86. [Article in Italian] doi: 10.1002/alz.13846 (PMC11180928; doi:10.1002/alz.13846)
Supplement: Supplementary file 6 — Supporting Information [file ALZ-20-3972-s005.docx]

**Supplemental material 6: Univariate meta-regression on hazard ratios for dementia incidence per one-point increase in LIBRA**

| **Moderator** | **Model 2** | | **Model 3** | |
| --- | --- | --- | --- | --- |
|  | **Exp (b)** | **P value** | **Exp (b)** | **P value** |
| Over entire follow-up period | | | | |
| Proportion female | 0.999 | .305 | 0.999 | .453 |
| Median age | **0.996** | **.011** | **0.994** | **.001** |
| Gross-domestic product per capita of country | 1.000 | .246 | 1.000 | .603 |
| Continent Europe | Reference | Reference | Reference | Reference |
| North America | 1.014 | .583 | 1.009 | .798 |
| Africa | 1.008 | .788 | 0.989 | .770 |
| Asia | **1.056** | **.011** | 1.052 | .076 |
| Median follow-up time | 1.003 | .254 | 1.006 | .086 |
| Number of available LIBRA factors | 1.008 | .268 | 1.010 | .195 |
| Early follow-up (within the first 5 years of follow-up) | | | | |
| Proportion female | 0.998 | .293 | 0.998 | .289 |
| Median age | 0.996 | .075 | **0.991** | **.003** |
| Gross-domestic product per capita of country | 1.000 | .632 | 1.000 | .809 |
| Continent Europe | Reference | Reference | Reference | Reference |
| North America | 1.018 | .670 | 1.034 | .517 |
| Africa | 1.011 | .823 | 0.987 | .822 |
| Asia | 1.041 | .248 | 1.051 | .196 |
| Number of available LIBRA factors | 1.013 | .197 | 1.018 | .073 |
| Late follow-up (at least 5 years after baseline) | | | | |
| Proportion female | 1.000 | .823 | 1.001 | .540 |
| Median age | 0.999 | .500 | 1.000 | .938 |
| Gross-domestic product per capita of country | 1.000 | .426 | 1.000 | .277 |
| Continent Europe | Reference | Reference | Reference | Reference |
| North America | 0.979 | .644 | 0.941 | .247 |
| Asia | 1.052 | .324 | 1.032 | .500 |
| Number of available LIBRA factors | 0.991 | .449 | 0.987 | .270 |

NOTE. Model 2: controlled for age, sex and years of formal education; Model 3: Model 2 + socioeconomic position. Gross-domestic product of the country was used as continuous measure for the income level of the country where the cohort was based. Values were retrieved from the World Bank (gross domestic product per capita per country in US dollars in 2002). Abbreviations: LIfestyle for BRAin health (LIBRA)
