# Supplementary material for: Lifestyle and incident dementia: A COSMIC individual participant data meta‐analysis
Source: Alzheimers Dement. 2024 Apr 27;20(6):3972–86. [Article in Italian] doi: 10.1002/alz.13846 (PMC11180928; doi:10.1002/alz.13846)
Supplement: Supplementary file 7 — Supporting Information [file ALZ-20-3972-s006.docx]

**Supplementary material 7: Sensitivity analysis without the Gothenburg H70 study**

Overall: hazard ratio (HR (95%CI)) for dementia per one-point increase in LIfestyle for BRAin health (LIBRA) score

| LIBRA score | Model 1, HR (95%CI) | Model 2, HR (95%CI) | Model 3, HR (95%CI) |
| --- | --- | --- | --- |
| Continuous  (per 1 point increase) | **1.07 (1.05 – 1.10)** | **1.06 (1.04 – 1.08)** | **1.05 (1.03 – 1.08)** |
| Lowest tertile | Reference | Reference | Reference |
| Middle tertile | **1.20 (1.03 – 1.40)** | 1.14 (0.99 – 1.32) | 1.08 (0.90 – 1.30) |
| Highest tertile | **1.44 (1.25 – 1.67)** | **1.32 (1.17 – 1.51)** | **1.28 (1.10 – 1.49)** |

NOTE. Model 1 controlled for age (time scale). Model 2 (main model): Model 1 + sex and years of formal education. Model 3: Model 2 + socioeconomic position. Abbreviations: hazard ratio (HR), confidence interval (CI), LIfestyle for BRAin health (LIBRA)

Stratified analysis: HR (95%CI) for dementia per one-point increase in LIBRA score

*By sex*

|  | Female | Male | Group differences |
| --- | --- | --- | --- |
| Model 1 | **1.065 (1.040-1.090)** | **1.073 (1.032-1.115)** | p=.738 |
| Model 2 | **1.054 (1.029-1.080)** | **1.065 (1.026-1.106)** | p=.647 |

NOTE. Model 1 controlled for age (time scale). Model 2 (main model): Model 1 + years of formal education

*By age*

|  | Up to 75 years old | 76 or older | Group differences |
| --- | --- | --- | --- |
| Model 1 | **1.094 (1.067-1.122)** | **1.056 (1.030-1.083)** | **p=.048** |
| Model 2 | **1.076 (1.048-1.105)** | **1.046 (1.022-1.070)** | p=.111 |

NOTE. Model 1 controlled for age (time scale). Model 2 (main model): Model 1 + sex and years of formal education

*By years of formal education*

|  | <6 years | 6-11 years | ≥ 12 years | Group differences |
| --- | --- | --- | --- | --- |
| Model 1 | **1.079 (1.051-1.108)** | **1.047 (1.010-1.085)** | **1.054 (1.002-1.109)** | p=.373 |
| Model 2 | **1.075 (1.047-1.104)** | **1.046 (1.009-1.086)** | **1.064 (1.005-1.126)** | P=.509 |

NOTE. Model 1 controlled for age (time scale). Model 2 (main model): Model 1 + sex

*By socioeconomic position (SEP)*

|  | Low | Intermediate | High | Group differences |
| --- | --- | --- | --- | --- |
| Model 1 | **1.045 (1.011-1.080)** | **1.074 (1.024-1.126)** | **1.067 (1.024-1.112)** | p=.582 |
| Model 2 | **1.044 (1.011-1.079)** | **1.065 (1.014-1.118)** | **1.057 (1.013-1.104)** | p=.782 |

NOTE. Model 1 controlled for age (time scale). Model 2 (main model): Model 1 + sex and years of formal education

*By geographical location*

|  | Africa | North America | Europe | Asia | Group differences |
| --- | --- | --- | --- | --- | --- |
| Model 1 | **1.062 (1.009–1.118)** | **1.065 (1.009-1.123)** | **1.048 (1.018-1.079)** | **1.119 (1.085-1.155)** | **.022** |
| Model 2 | 1.060 (0.981-1.144) | **1.055 (1.001-1.112)** | **1.040 (1.011-1.069)** | **1.100 (1.065-1.137)** | **.078** |

NOTE. Model 1 controlled for age (time scale). Model 2 (main model): Model 1 + sex and years of formal education

Meta-regression

Univariate meta-regression on HRs for dementia incidence per one-point increase in the LIBRA index. Model 2 controlled for age, sex and years of formal education. Model 3: Model 2 + socioeconomic position.

|  | **Model 2** | | **Model 3** | |
| --- | --- | --- | --- | --- |
| **Moderator** | **Exp (b)** | **P value** | **Exp (b)** | **P value** |
| Proportion female | 0.998 | .294 | 0.998 | .343 |
| Median age | **0.996** | **.014** | **0.994** | **.001** |
| Gross-domestic product per capita of country | 1.000 | .252 | 1.000 | .526 |
| Continent |  |  |  |  |
| Europe | Reference | Reference | Reference | Reference |
| North America | 1.016 | .552 | 1.016 | .644 |
| Africa | 1.011 | .725 | 0.996 | .909 |
| Asia | **1.058** | **.014** | **1.059** | **.043** |
| Median follow-up time | 1.004 | .214 | 1.007 | .097 |
| Number of available LIBRA factors | 1.008 | .298 | 1.010 | .236 |
